# Functional MRGPRX2 expression on peripheral blood-derived human mast cells increases at low seeding density and is suppressed by interleukin-9 and fetal bovine serum

**DOI:** 10.3389/fimmu.2024.1506034

**Published:** 2024-12-13

**Authors:** Toon Ieven, Janne Goossens, Willem Roosens, Anne-Charlotte Jonckheere, Jonathan Cremer, Ellen Dilissen, Rune Persoons, Lieven Dupont, Rik Schrijvers, Peter Vandenberghe, Christine Breynaert, Dominique M. A. Bullens

**Affiliations:** ^1^ KU Leuven Department of Microbiology, Immunology and Transplantation, Allergy and Clinical Immunology Research Group, Leuven, Belgium; ^2^ Division of General Internal Medicine, Allergy and Clinical Immunology, UZ Leuven, Leuven, Belgium; ^3^ KU Leuven Department of Chronic Diseases and Metabolism, Laboratory of Respiratory Diseases and Thoracic Surgery (BREATHE), Leuven, Belgium; ^4^ Division of Respiratory Diseases, UZ Leuven, Leuven, Belgium; ^5^ KU Leuven Department of Human Genetics, Laboratory for Genetics of Malignant Disorders, KU Leuven, Leuven, Belgium; ^6^ Division of Hematology, UZ Leuven, Leuven, Belgium; ^7^ Division of Pediatrics, UZ Leuven, Leuven, Belgium

**Keywords:** human, blood, mast cell, culture, MRGPRX2, interleukin-9, serum, ketotifen

## Abstract

Primary human mast cells (MC) obtained through culturing of blood-derived MC progenitors are the preferred model for the *ex vivo* study of MRGPRX2- *vs.* IgE-mediated MC activation. In order to assess the impact of culture conditions on functional MRGPRX2 expression, we cultured CD34^+^-enriched PBMC from peripheral whole blood (PB) and buffy coat (BC) samples in MethoCult medium containing stem cell factor (SCF) and interleukin (IL)-3, modified through variations in seeding density and adding or withholding IL-6, IL-9 and fetal bovine serum (FBS). Functional expression of MRGPRX2 was assessed after 4 weeks via flow cytometry. We found similar proportions of CD34^+^ MC-committed progenitors in BC and PB. Higher seeding densities (≥ 1x10^5^ cells/mL) and exposure to IL-9 and FBS suppressed functional MRGPRX2 expression at 4 weeks, while leaving MC yield largely unaffected. IL-6 had no impact on MRGPRX2 expression. MRGPRX2-expressing MC upregulated CD63 upon stimulation with polyclonal anti-IgE, substance P and compound 48/80 at 4 weeks. Ketotifen and dasatinib but not cromolyn sodium inhibited both IgE- and MRGPRX2-dependent pathways. Our results confirm the feasibility of functional MC activation studies on PB-derived MC after a short 4-week culture and highlight the impact of culture conditions on functional MRGPRX2 expression.

## 1 Introduction

Mast cells (MC) are tissue-resident mononuclear granulocytes of myeloid origin involved in host defense and tissue homeostasis (1, 2). Insights gained from murine models indicate that MC ontogeny is complex, with separate MC populations derived from distinct embryonic stem cell subsets (3). Nevertheless, it is generally accepted that most MC in adult humans originate from bone marrow (BM) CD34^+^ progenitors that migrate through blood towards target tissues where they differentiate into mature MC under the influence of stem cell factor (SCF) (4). MC and basophils strongly express high-affinity IgE receptors (FcϵRI). While most research focuses on their role as effectors of IgE-mediated (type I) hypersensitivity, recent insights imply MC in a wide range of non-allergic diseases, including chronic spontaneous urticaria, irritable bowel syndrome, atherosclerosis, auto-immune/auto-inflammatory diseases and malignancy (1–7).

MC possess a uniquely broad spectrum of receptors, allowing them to respond to a wide range of triggers (8). MC degranulation has long been known to occur independently of FcϵRI cross-linking upon contact with certain cationic peptides, synthetic compounds and drugs (9–11). In 2015, McNeil et al. discovered the Mas-related G-protein-coupled receptor member X2 (MRGPRX2) to be the target of these so-called MC secretagogues (12). Since then, evidence has emerged in support of MRGPRX2 as the “missing link” in so-called pseudo-allergic or anaphylactoid reactions characterized by symptoms of type I hypersensitivity without detectable allergen-specific IgE (13–15). The list of MRGPRX2 agonists with potential clinical relevance is rapidly expanding and includes synthetic drugs, insect venom components and herbal compounds (13–18). In addition, MRGPRX2 is believed to play a role in the pathogenesis of MC-related disorders, including rosacea, atopic dermatitis, non-histaminergic pruritus, irritable bowel syndrome and mediator symptoms in mastocytosis (18–21).

Due to a lack of biomarkers for distinguishing MRGPRX2- from IgE-mediated MC activation, evidence on its clinical relevance in human disease remains indirect, inferred from clinical observations or derived from preclinical models (14). Such models rely on the availability of sufficient amounts of MRGPRX2-expressing cells to allow *in vitro* characterization and stimulation. Since murine Mrgprb2 shares only 53% sequence identity with human MRGPRX2, translatability to humans is limited (22). MRGPRX2-transfected non-MC cell lines (e.g. HEK293) can screen compounds for MRGPRX2-activating potential but cannot offer conclusive insights on MC activation (22–24). As such, human cells that natively express MRGPRX2 are the preferred study model. However, their use is subject to several pitfalls. First, primary human MC are low-abundant and absent from the bloodstream (25). Furthermore, their phenotype differs based on micro-environmental factors as skin MC express both tryptase and chymase (MC_TC_) and higher levels of MRPGRX2 whereas lung MC (MC_T_) have lower chymase and MRGPRX2 expression (1, 4, 5, 26). Second, while basophils are more easily accessible, they lack MRGPRX2 expression in resting circumstances (27). Third, though immortalized human MC lines such as LAD-2 and HMC-1 are a source of human MC, they vary in MRGPRX2 expression and respond differently to MRGPRX2-mediated activation compared to primary MC (28). Lastly, since genetic polymorphisms in MRGPRX2 likely determine the risk of MRGPRX2-mediated hypersensitivity, diagnosis of such reactions (i.e. direct mast cell activation testing or dMAT) should ideally use MC sharing the patient’s genetic background (25, 29–31).

Methods for generating large quantities of mature primary human MC have existed for decades, based on induced pluripotent stem cells or progenitor cells obtained from peripheral blood, BM, cord blood (CB) or fetal liver tissue cultured in specific media supplemented with SCF and other growth factors for several weeks to months (25, 32). Many groups have evaluated the impact of varying cytokine combinations and culturing conditions on yield, protease content and functional FcϵRI expression. However, evidence on the effects of culture conditions on MRGPRX2 expression is limited (27, 33–36).

We adapted previously published short (3- to 4-week) protocols for primary human MC differentiation starting from readily accessible peripheral whole blood (PB) and buffy coat (BC) concentrates, aiming to investigate the impact of culture conditions and growth factor combinations on functional MRGPRX2 expression and assess whether the obtained MC can be used for pharmacological modulation of IgE- and MRGPRX2-dependent MC activation pathways.

## 2 Methods

See **Supplementary Methods** section in the **Supplementary Data Sheet 1** for details.

### 2.1 Sampling and ethics

PB samples (± 80 mL) were obtained on lithium heparin anticoagulant from consenting healthy adults in accordance with protocols approved by the local institutional review board (Ethics Committee UZ/KU Leuven, S65209 and S62076). BC concentrates (± 40 mL per donor) were obtained from Red Cross Belgium as rest products after blood donation (± 500 mL per donor) on citrate-phosphate-dextrose. PB was processed within 4 hours and BC within 24 hours after donation.

### 2.2 Mast cell cultures

**Figure 1:**
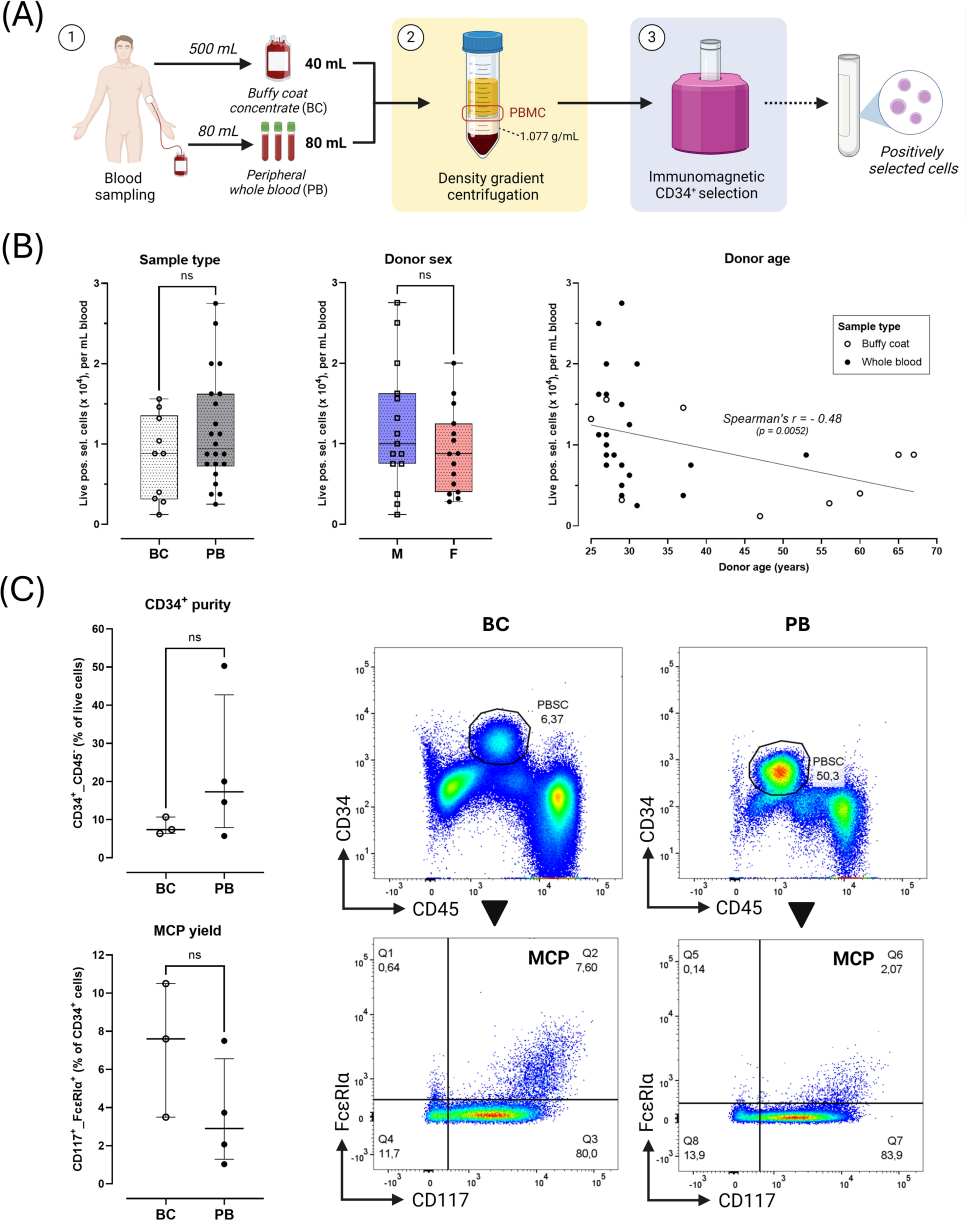
Starting sample characteristics. **(A)** Overview of sampling and processing. 1) Buffy coat concentrates (BC, 40 mL) were acquired as rest products after blood donation through centrifugation of 500 mL peripheral whole blood. Peripheral whole blood (PB, 80 mL) was directly drawn in vacuum tubes on lithium-heparin anticoagulant. 2) Peripheral blood mononuclear cells (PBMC) were collected from the interphase after density gradient centrifugation. 3) PBMC were enriched for circulating peripheral blood CD34^+^ stem cells (PBSC) through positive immunomagnetic selection. The positively selected cell fraction was analyzed via flow cytometry or seeded into cultures. **(B)** Yield of positively selected cells normalized per mL initial donor blood volume according to sample type (BC [n=10] vs. PB [n=22]), donor sex (male, M [n=15] vs. female, F [n=15]) and donor age (with linear regression line). **(C)** Flow cytometric characterization of PBMC fractions obtained after step 3 (BC [n=3]), PB [n=4] samples). Black arrows indicate directionality of flow cytometric gating. Quadrant gating was applied based on FMOs. Results shown as median ± IQR. Plots show representative gating of CD34^+^ PBMC (top panels) and CD34^+^_CD117^hi^_FcϵRIα^hi^ mast cell-committed progenitors (MCP, Q2 gate) for one PB donor (CO1) and one BC donor (RK9). Horizontal error bars indicate Mann-Whitney U unpaired comparisons between groups (ns = not significant, p > 0,05).

PB mononuclear cells (PBMC) were isolated using density gradient centrifugation over Lymphoprep™ (1.077 g/L; Stemcell Technologies). CD34^+^ cell selection was performed through 5 cycles with the EasySep™ Human CD34 Positive Selection Kit II (Stemcell Technologies, Vancouver, Canada). Positively selected cells were suspended in 1 mL Iscove’s modified Dulbecco’s medium (IMDM; Gibco ThermoFisher, Waltham, Mass, U.S.) and counted (**Figure 1A**).

A 4-week culture protocol was adapted and modified from previous reports (**Supplementary Figure S1A**) (37–40). Starting medium consisted of serum-free methylcellulose medium (Methocult™, Stemcell Technologies) mixed in a 4:1 ratio with IMDM containing penicillin-streptomycin (P/S; 100 U/mL-100 µg/mL), β-mercaptoethanol (55 µM), human low-density lipoprotein (10 µg/mL, Stemcell Technologies), recombinant human SCF (100 ng/mL; Miltenyi Biotec, Bergisch Gladbach, Germany) and recombinant human interleukin (IL)-3 (50 ng/mL; PeproTech, Cranbury, NJ, U.S.). Cells were added to IMDM at 5X final desired seeding density. Cells were distributed across 6- or 24-well plates (1.1 mL/well) and cultured at 37°C and 5% CO_2_. Nourishing medium was added every 3-4 days through gentle layering of 0.3 mL IMDM with bovine serum albumin (BSA; 0.1%), P/S (100 U/mL), SCF (20 ng/mL), insulin-transferrin-selenium (1x; Gibco) and various cytokines (at 20 ng/mL). After 3 weeks, cells were transferred to resting medium (RM) containing IMDM, SCF (20 ng/mL) and P/S (P/S; 100 U/mL-100 µg/mL) at a density of 0.5x10^6^/mL for 3-7 days, followed by final analysis.

Variations in conditions included low (1-4x10^4^/mL) or high (1-2x10^5^/mL) seeding densities and adding/withholding recombinant human IL-6 (continuously; 50 ng/mL in starting medium; PeproTech), IL-3 (week 1 or continuously), IL-9 (week 2-3; 20 ng/mL; PeproTech) or FBS 5% (week 4).

### 2.3 Stimulation and inhibition experiments

Stimulation/inhibition experiments were performed on 2.5-5x10^4^ cells/condition, suspended in 90 µL HEPES buffer (41). All reagents were acquired from Sigma-Aldrich (St. Louis, MO, U.S.) and stored at 100X (inhibitors) or 10X (stimulants) working solutions. Inhibitors (1 µL of working solution) were added to cells prior to stimulation for 15’ at 37°C in a water bath and included dasatinib (das; 1 µM final concentration), ketotifen (ket; 100 µM) and cromolyn sodium (CS; 1 mM). MRGPRX2-mediated stimulation of unsensitized cells was performed with either 10 µL substance P (SP; 0.1 mg/mL or 74.2 µM) or compound 48/80 (C48/80; 3.12 µg/mL or 0.62 µM) for 20’ at 37°C. IgE-mediated stimulation was performed after overnight passive sensitization with human serum followed by washing and stimulation with 10 µL polyclonal goat anti(a)-IgE (1 µg/mL) for 60’ at 37°C. Stimulation was followed by resting on ice for 5’, washing and staining. CD63-expression was used as a proxy for MC activation (41, 42).

### 2.4 Immunophenotyping

Dead cells were stained using Fixable Viability Dye (FVD) eFluor™ 780 (eBioscience, San Diego, Calif., U.S.) followed by washing, staining in the dark for 25’ at 4°C with various fluorochrome-conjugated antibody panels (see **Supplementary Table S1**), washing and fixation in paraformaldehyde (PFA) 1% and storage at 4°C prior to acquisition on an LSR Fortessa flow cytometer equipped with FACSDiva software (BD, Franklin Lakes, NJ, U.S.). Fluorochrome-conjugated antibodies were acquired from BioLegend (San Diego, Calif., U.S.). Final analysis was performed using FlowJo v10.8.1 for Windows (BD, San Jose, Calif., U.S.). Full sets of compensation controls (UltraComp eBeads™ Plus; Invitrogen; Waltham, Mass., U.S.) and appropriate fluorescence-minus-one (FMO) controls were included for each experiment. Panels included a progenitor (CD45, CD34, CD117, FcϵRIα; see **Supplementary Figure S2** for gating strategy), differentiation/activation (CD117, CD203c, MRGPRX2, FcϵRIα and CD63; **Supplementary Figure S3**) and intracellular protease expression panel (tryptase, chymase) (**Supplementary Figure S4**).

### 2.5 Cytospins and cytochemical staining

For microscopic evaluation of cultured cells, 50 µL medium containing 50.000 cells were layered onto glass slides using a CytoSpin III centrifuge for 4’ at 800 rpm (Shandon Scientific, Astmoor, U.K.). Monolayers were stained with RAL Diff-Quick™ (CellaVision, Martillac, France) or fixed with Clarke’s fixative (75% ethanol, 25% acetic acid) for 10’ followed by indirect rinsing with deionized water and staining with 2-3 droplets of toluidine blue dye (Sigma-Aldrich) for 20’ at RT. Stained slides were rinsed, air-dried and provided with cover slips fixed with xylene.

### 2.6 Statistical analysis

Graphs show medians with interquartile ranges (IQR). Comparisons were performed with the Mann-Whitney U test (unpaired data) or Wilcoxon matched-pairs signed rank test (paired data). Correlation between continuous variables was assessed using Spearman’s r. A p-value < 0.05 was considered statistically significant for all tests. GraphPad Prism v10.2.3 for Windows (Dotmatics, Boston, Mass., U.S.) was used for statistical analyses and figure generation. Illustrations were created using BioRender (Toronto, Ont., Canada).

## 3 Results

### 3.1 Buffy coat concentrates and whole blood have similar mast cell progenitor content per unit of initial blood volume

In total, 10 BC samples from 10 donors (RK1-10) and 22 PB samples from 13 healthy donors (CO1-13) were obtained after written informed consent (see **Supplementary Table S2** for an overview of donors).

Live PBMC were counted after density gradient centrifugation and CD34^+^ cell enrichment (see **Figure 1A** for workflow). The median amount of PBMC and positively selected cells retrieved per mL BC was significantly higher vs. PB (20x10^6^ vs. 1.8x10^6^ and 11x10^4^ vs. 0.94x10^4^ cells/mL, respectively; p < 0.0001) (**Supplementary Figure S5**). This difference was no longer apparent after normalizing cell counts to initial blood volume (**Figure 1B**). Cell retrieval was similar in male and female donors (**Figure 1B**). While PBMC retrieval was independent of donor age (**Supplementary Figure S6**), cell yield after positive selection correlated negatively with donor age (Spearman’s r -0.48, p < 0.01; **Figure 1B**).

In 3 BC and 4 PB samples, PBMC fractions obtained after immunomagnetic selection were assessed for CD34^+^ enrichment efficiency (**Figure 1C**). Median CD34^+^ purity in the positively selected fraction was 10.7% (range 6.4 - 50.3%). No CD34^+^ cells were detected in rest fractions. The median percentage of MC-committed progenitors (MCP; CD34^+^_CD117^hi^_FcϵRIα^hi^) within the CD34^+^ population was 3.7 (range 2.1 – 10.5%). No significant differences in CD34^+^ or MCP purity were observed between BC and PB samples (**Figure 1C**).

### 3.2 Phenotypically mature mast cells are obtained after 4 weeks of culture with significant inter- and intra-donor variability

**Figure 2:**
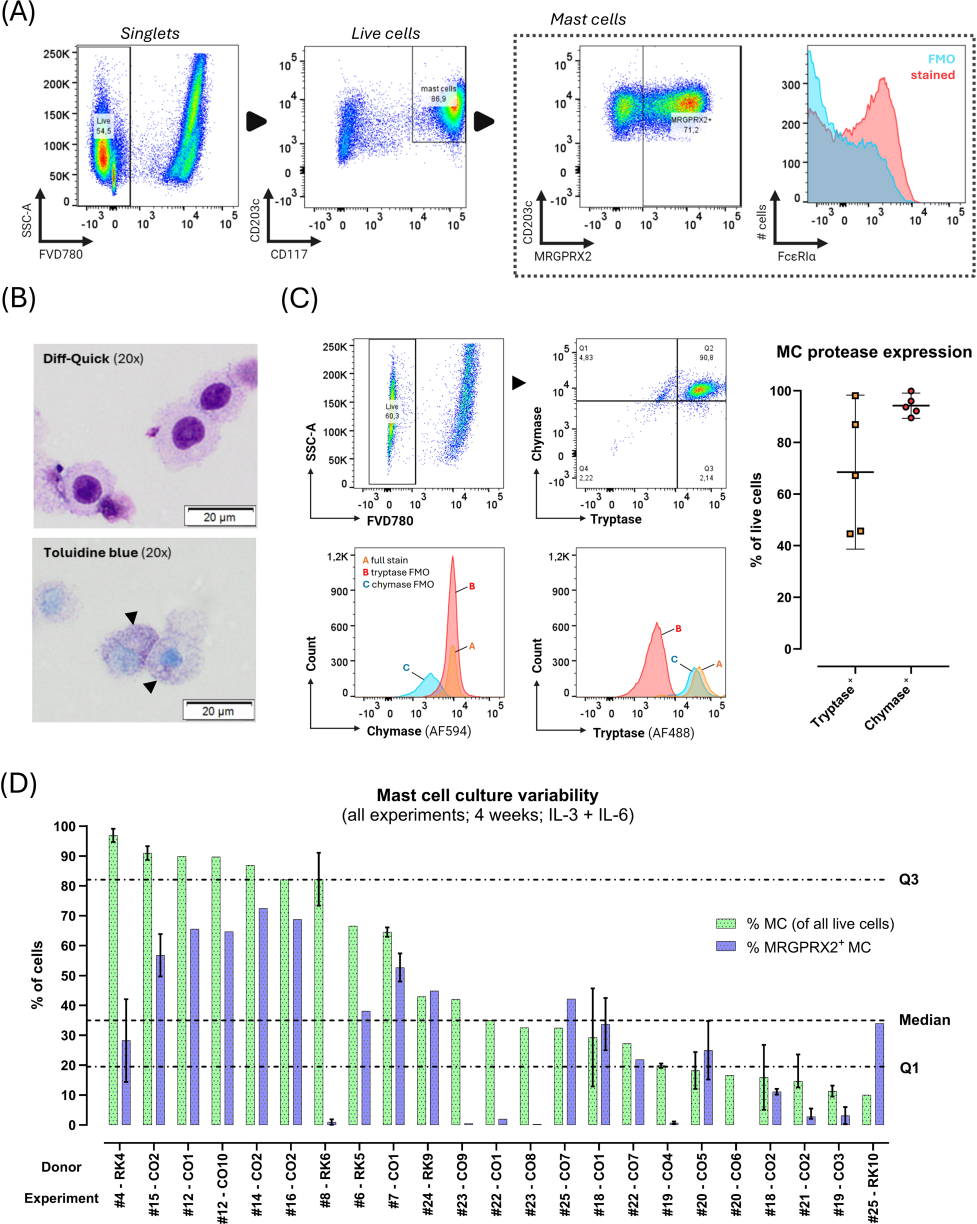
Phenotype of blood-derived primary human mast cells (MC) after 4 weeks culture with stem cell factor, IL-6 and IL-3 (first week only). **(A)** MC immunophenotype assessed with flow cytometry (donor CO2 shown). Black arrows indicate directionality of gating. First, live (FVD780^-^) cells were selected within singlets, and CD117^hi^_CD203^+^ MC were selected within live cells; next, MRGPRX2^+^ cells were identified among live MC. Due to presence of autofluorescence in the FcϵRIα channel (see blue FMO population), the %FcϵRIα^+^ MC could not be reliably determined in the fully stained (red population) sample. **(B)** Light microscopy of cytospins stained with Diff-Quick™ (top) or toluidine blue (bottom). Toluidine blue staining reveals dark-purple metachromatic granules in the cytoplasm (black arrowheads) (donor CO2 shown). **(C)** Intracellular MC protease expression assessed through flow cytometry in live cells after 4 weeks of culture. Top row shows the flow cytometric gating strategy for intracellular proteases on a single representative donor sample (CO2). Quadrant gates (top right panel) were set on all live cultured cells (FVD780^-^) based on FMO’s (bottom panels). The plot on the right shows the median (± IQR) % of live tryptase^+^ and chymase^+^ cells after 4 weeks of culture (n=5 cultures). **(D)** Results of repeated culture experiments (n=23) from various donors (n=15) cultured under similar circumstances for 4 weeks with IL-3 (week 1 only) and IL-6 (continuously). Y-axis indicates %MC (CD117^hi^_CD203c^+^ as % of all live cells; green bars) and %MRGPRX2^+^ MC (as % of total MC; blue bars) obtained at the end of the culture, ordered from highest to lowest MC purity. X-axis indicates the donor and experiment number, respectively. For donors with multiple culture replicates under the same circumstances during the same culture run, results are shown as median with IQR (vertical error bars). Horizontal dotted lines indicate global median and IQR of %MC.

Flow cytometric assessment of cultured cells after 4 weeks revealed the presence of differentiated MC through co-expression of CD117 and CD203c with/without MRGPRX2 on a subset of cultured cells of varying size (**Figures 2A, D**). Cytochemical staining revealed presence of large (± 20 µM diameter), round, mononuclear cells containing metachromatic cytoplasmic granules, confirming a mature MC phenotype (**Figure 2B**). Intracellular MC protease expression was assessed in selected experiments (n=5), revealing a median tryptase expression of 65.4% (range 44.7 – 99.6%) and median chymase expression of 92.3% (range 88.7 – 99.2%) (**Figure 2C**). The proportion of protease-expressing cells exceeded the proportion of CD117^+^_CD203c^+^ cells.

The median yield of total viable cells after 4 weeks of culture was 7.4x10^6^ (range 0.3 – 32x10^6^), starting from a median of 0.9x10^6^ initially seeded cells (range 0.2 – 7.8x10^6^) amounting to a median ratio of total harvested to seeded cells of 8.3.

**Figure 3:**
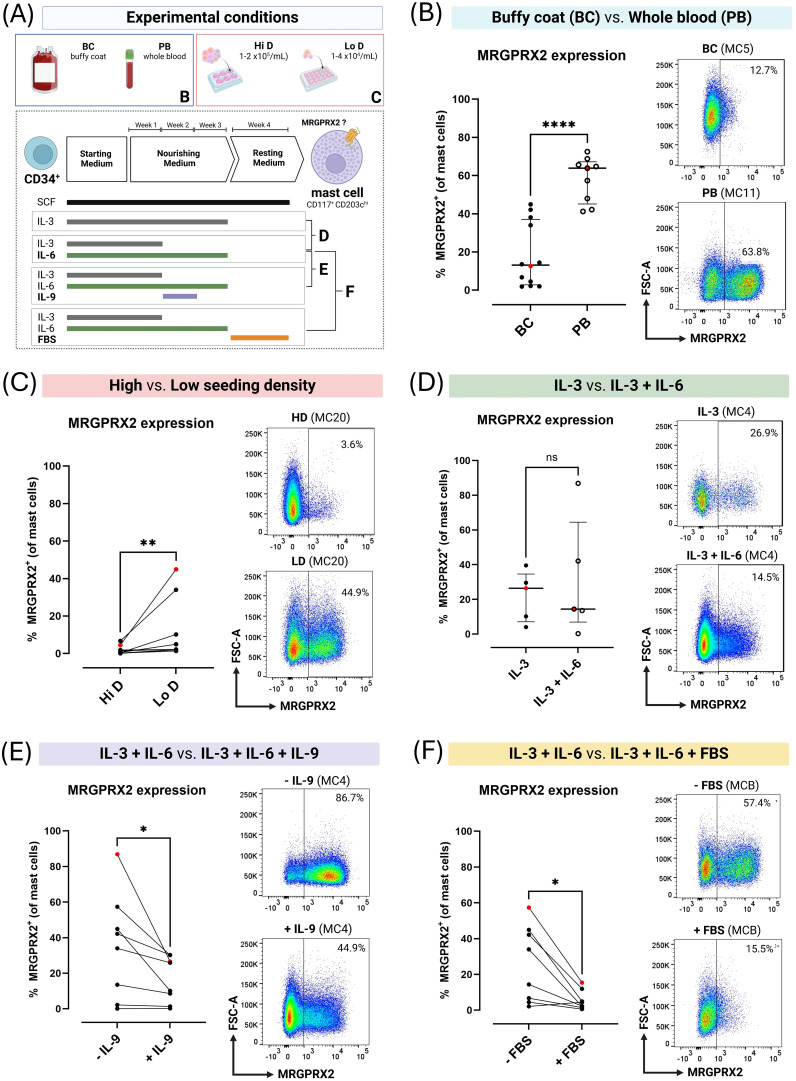
MRGPRX2 expression on blood-derived primary human mast cells (MC) cultured for 4 weeks in MethoCult under varying culture conditions. **(A)** Overview of experimental culture conditions. **(B)** Unpaired comparison between buffy coat concentrate (BC)-derived (n=12) and peripheral whole blood (PB)-derived (n=9) MC cultures. **(C)** Paired comparison between BC-derived MC cultures (n=7) seeded at high density (Hi D; 1-2x10^5^ cells/mL) or low density (Lo D; 1-4x10^4^ cells/mL). **(D)** Unpaired comparison between MC cultured in continuous presence of interleukin (IL)-3 or IL-3 during week 1 and IL-6 continuously (n=5). **(E)** Paired comparison between MC cultured in IL-3 and IL-6 or IL-3 and IL-6 with addition of IL-9 during week 2 (n=8). **(F)** Paired comparison between MC cultured with or without fetal bovine serum (FBS) during week 4 (n=8). Each dot corresponds to a single donor (in single replicate). Red dots correspond to the flow cytometry plots shown. Error bars show median and interquartile ranges for unpaired data. For paired data, cultures obtained from the same donor during the same experiment are connected with lines. Horizontal lines indicate comparisons between groups using the Mann-Whitney U test for unpaired data and the Wilcoxon matched-pairs signed rank test for paired data. Pairwise comparisons were made between cultures derived from the same donor during the same culture run. ns = not significant (p > 0.05), * = p < 0.05, ** = p < 0.01, **** = p < 0.001.

Remarkable inter- and intra-donor variability was observed. Across 26 separate cultures with the same experimental parameters on samples from 15 donors, median MC purity was 36% (range 10% - 96.9%), and median MC MRGPRX2 expression was 23.5% (range 0% - 72.5%) (**Figure 2D**). Culture yields not only differed between donors but also within donors. BC-derived cultures yielded significantly lower MRGPRX2-expression compared to PB-derived cultures, without differences in MC purity (**Figure 3B**; **Supplementary Figure S6**).

### 3.3 Seeding density influences mast cell culture purity, MRGPRX2 expression and culture yield

The density at which positively selected cells were seeded was theorized to affect culture outcomes, potentially explaining differences between BC- and PB-derived cultures. This was supported by paired experiments with PB-derived cultures where low seeding densities resulted in a significantly lower median MC purity compared to high seeding densities (32.2% vs. 69.8%; p = 0.0195; **Supplementary Figure S1**) but significantly higher rates of MRGPRX2 expression (2.2% vs. 0.6%; p < 0.005; **Figure 3C**). When including both (unpaired) PB- and BC-derived cultures, lower seeding densities also appeared to result in greater progenitor expansion with a median ratio of total harvested to seeded cells of 10.5 in low-density cultures (range 2.3 – 45.7) vs. 2.7 in high-density cultures (range 0.9 – 22.5) (p = 0.0089) (**Supplementary Figure S7**).

### 3.4 Short-term exposure to IL-9 or fetal bovine serum negatively impacts MRGPRX2 expression

As varying combinations of growth factors are reported to differentially impact the phenotype of cultured human MC, we evaluated the impact of IL-6, IL-9 and FBS on culture outcomes focusing on MRGPRX2 expression at 4 weeks (**Figure 3A**).

Continuous addition of only IL-3 vs. continuous addition of IL-6 with IL-3 in the first week did not result in a significant difference in MRGPRX2 expression after 4 weeks. In contrast, MC purity seemed to be higher compared to IL-3 alone (median 94.7 vs. 71%; p = 0.0317; **Figure 3D**; **Supplementary Figure S1**).

Paired cultures exposed to IL-6 (continuous) and IL-3 (week 1) with or without IL-9 (week 2) revealed addition of the latter to significantly decrease MRGPRX2 expression (18% with vs. 38.1% without IL-9; p = 0.0156) while not affecting MC purity (**Figure 3E**; **Supplementary Figure S1**). Similarly, adding FBS during the final week of culture almost completely abrogated MRGPRX2 expression (3.4% with vs. 24.2% without FBS; p = 0.0156) without affecting MC purity (**Figure 3F**; **Supplementary Figure S1**).

### 3.5 Mast cells obtained after 4 weeks express functional MRGPRX2- and IgE-dependent activation pathways

**Figure 4:**
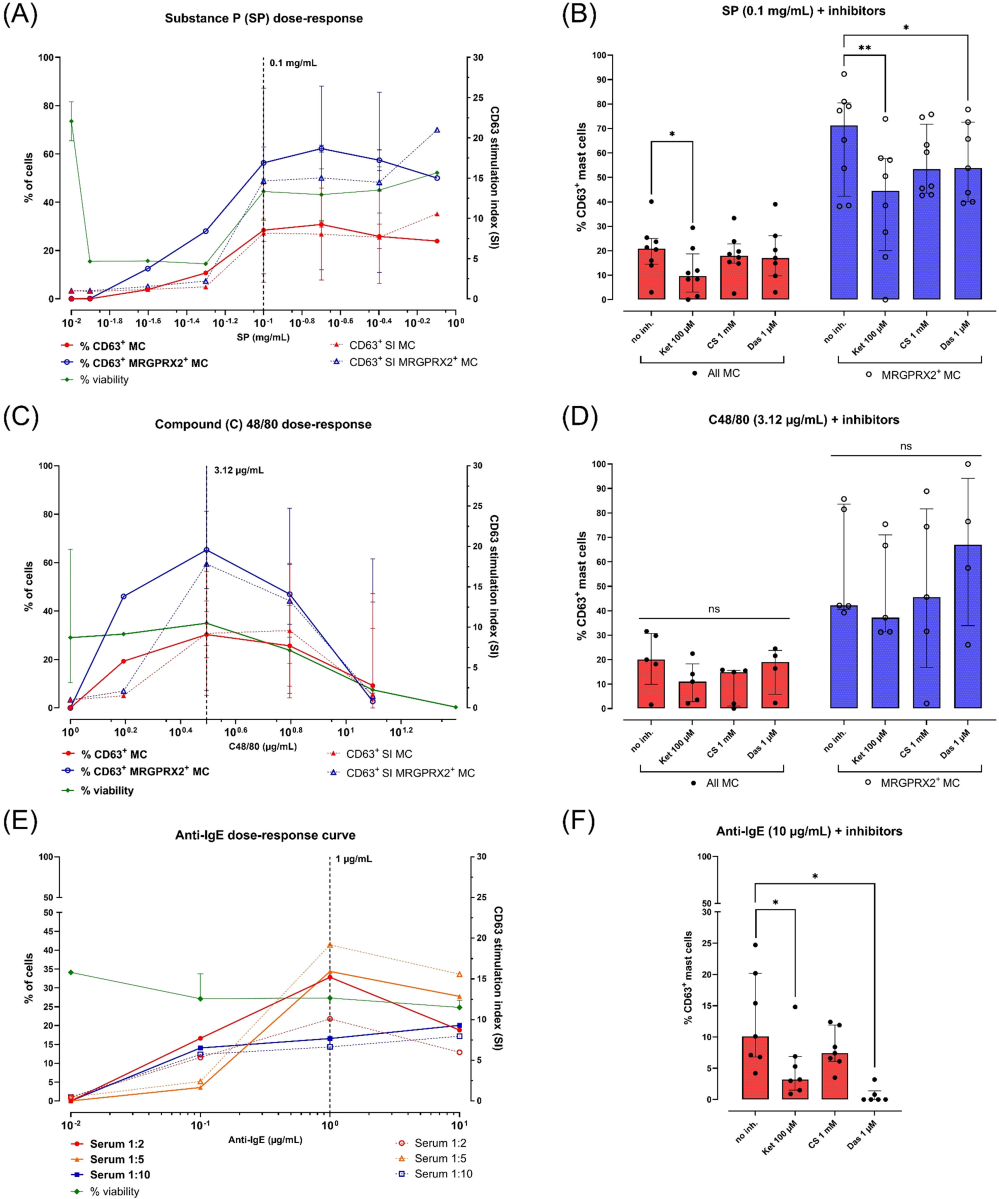
Responses of peripheral blood-derived primary human mast cells (MC) to various stimuli and inhibitors. All MC were obtained after 4 weeks of culture in MethoCult with IL-3 (week 1) and IL-6 (continuous). MC activation is reported as % CD63+ live MC corrected for spontaneous activation (left y-axes, continuous lines) or as CD63 stimulation indices (SI; right y-axes, dotted lines). MC viability (% FVD780^-^ cells) is indicated for each concentration of stimulus (left y-axes, continuous green line). Each dot represents a single donor (in single replicate). The number (n) of donors used is indicated between brackets. **(A)** CD63 dose-response curves after stimulation with substance P (SP) 0.0125 – 0.8 mg/mL (n=2, median + range), **(B)** MC activation by SP 0.1 mg/mL after preincubation with buffer or inhibitors (n=8, median + IQR). **(C)** CD63 dose-response curves after stimulation with compound 48/80 (C48/80) 1.56 – 100 µg/mL (n=3, median + range), **(D)** MC activation by C48/80 3.12 µg/mL after preincubation with buffer or inhibitors (n=5 donors, median + IQR), **(E)** CD63 dose-responses curve after overnight sensitization with human serum (1:10, blue squares; 1:5, orange triangles; 1:2, red circles) and stimulation with polyclonal goat anti-human IgE 0.1-10 µg/mL (n=1), **(F)** MC activation by anti-IgE 10 µg/mL after overnight passive sensitization in human serum 1:5 and preincubation with buffer or inhibitors (n=4, median + IQR). Horizontal bars indicate pairwise comparisons between groups using the Wilcoxon matched-pairs signed rank test. ns = not significant (p ≥ 0.05), * = p < 0.05, ** = p < 0.01.

SP was tested at 7 concentrations (0.0125 – 0.8 mg/mL) in 2 separate cultures, inducing a CD63 dose-response curve peaking at 0.1 – 0.2 mg/mL (**Figure 4A**). The %CD63^+^ MC at 0.1 mg/mL was ± 2-fold higher in the MRGPRX2^+^ subset compared to the total MC population (56.3% vs. 28.5%).

C48/80 was also tested at 7 concentrations (1.56 – 100 µg/mL) in 4 separate MC cultures, inducing a dose-response curve peak at 3.12 µg/mL (**Figure 4C**). C48/80 concentrations above 3.12 µg/mL resulted in a steep drop in viability (i.e. %FVD780^+^ cells) in all 4 donors. Similar to SP, C48/80 induced a ± 2-fold higher CD63-expression at 3.12 µg/mL in the MRGPRX2^+^ subset compared to the total MC population (65.3% vs. 30.3%).

For anti-IgE stimulation, MC from a single donor were passively sensitized overnight at 37°C with 3 dilutions of human serum (1:10 up to 1:2). Stimulation with aIgE (0.1 – 10 µg/mL) resulted in dose-dependent CD63 expression reaching a peak at 1 µg/mL (**Figure 4E**). The strongest aIgE dose response was observed in MC sensitized at a 1:5 serum dilution (maximum of 34.4% CD63^+^ MC). Higher serum concentrations during passive sensitization did not affect MC viability.

As a proof-of-concept, a passive MAT was performed using serum of a yellow jacket venom (YJV)-allergic donor followed by stimulation with YJV at 0.01 – 10 µg/mL (**Supplementary Figure S8**). Cells passively sensitized with serum in a 1:5 ratio responded with a weak dose response up to a maximum of 9.6% CD63^+^ MC at 10 µg/mL YJV.

Stimulations were also performed in MC from the same donors cultured in absence or presence of FBS during the final week. As was expected based on MRGPRX2 expression, FBS exposure abrogated the ability of MC to respond to SP and C48/80 challenge while leaving IgE-dependent activation largely unaffected (**Supplementary Figure S9**).

### 3.6 Ketotifen partially suppresses MRGPRX2- and IgE-dependent mast cell activation

Effects of various MC-stabilizing compounds were tested after exposure to agonists at their optimal stimulatory concentrations (**Figures 4B, D, F** and **Supplementary Figure S10**).

Stimulation with SP 0.1 mg/mL was inhibited after preincubation with ketotifen 100 µM, resulting in a median 48.5% decrease in %CD63^+^ MC (p = 0.0156). CS 1000 µM had only a weak inhibitory effect (17%; p > 0.05). Dasatinib 1 µM demonstrated a very weak inhibitory effect (6.9%; p > 0.05), although this effect was more pronounced and significant within the MRGPRX2^+^ subpopulation (18.9%; p = 0.0321) (**Figure 4B** and **Supplementary Figure S10**). Surprisingly, no significant impact of inhibitors was observed on MC activation by C48/80 at 3.12 µg/mL (**Figure 4D** and **Supplementary Figure S10**).

IgE-dependent MC activation with anti-IgE 1 µg/mL was also significantly inhibited by ketotifen (median %CD63^+^ decrease of 65.6%; p = 0.0312) and dasatinib (median decrease of 100%; p = 0.0312). Preincubation with CS trended towards decreased CD63 expression, although this was not statistically significant (39.4%; p > 0.05) (**Figure 4F**; **Supplementary Figure S10**).

## 4 Discussion

As primary human MC are the preferred model for MRGPRX2-mediated hypersensitivity, it is crucial to identify parameters that affect MRGPRX2 expression during *ex vivo* MC differentiation, although data is still limited (25). Our work demonstrates that variations in the culture micro-environment can profoundly impact MRGPRX2 expression and that such factors should be taken into account when performing MRGPRX2-focused studies.

Seeding density was the first crucial factor. Many protocols use seeding densities above 1x10^5^/mL, coincidentally requiring larger PB volumes (38–40). In contrast, Saito et al. highlight the benefits of lower seeding densities (< 1x10^5^ cells/mL) as higher densities limit CD34^+^ self-renewal and increase formation of other cell types which can negatively influence MC development (37, 43, 44). We found a negative impact of higher seeding densities on final culture yield and MRGPRX2 expression. The latter might also explain the lower MRGPRX2 expression observed in BC-derived cultures which were initially seeded at higher densities. BC and PB are easily accessible MCP sources used in many recent MC culture protocols (28). BC are, in essence, concentrated PB samples, as was reflected in their similar CD34^+^ and MCP composition, albeit at higher quantities per unit of sample volume. This equivalence was further reflected in similar MC purities obtained in BC and PB-derived MC cultures at 4 weeks. These findings, as well as observations that PB-derived cultures result in higher MC yield and purity after 4 weeks compared to BM-derived cultures, support the use of PB (or BC) at lower seeding densities for short (4-week) MRGPRX2-focused MC cultures (35).

A second factor was IL-9 exposure. Previous studies on PB- and CB-derived MC reported that IL-9, a crucial cytokine in type 2 (IgE-dependent) immune responses, improves progenitor expansion and final yield during early culture phases without affecting final MC differentiation status (38, 45). Our results show for the first time that short-term IL-9 exposure negatively impacts MRGPRX2 expression in MC obtained after 4 weeks. IL-4, another major type 2 cytokine, has been shown to decrease MC proliferation and differentiation early on while enhancing functional FcϵRI expression during the late culturing phase (38, 46). A recent study by Babina et al. found that IL-4, synergistically with SCF, rapidly stimulated FcϵRI expression while partially suppressing MRGPRX2 expression in *ex vivo* cultured human skin MC (33). Whether an inverse relationship between IgE- and MRGPRX2-dependent MC activation pathways exists *in vivo* merits further investigation.

A third factor was the addition of FBS. Most protocols use serum-free conditions to promote progenitor expansion and improve MC yield, though some groups add FBS at later time points to increase both FcϵRI expression and histamine content (47). We found that addition of FBS 5% to the medium during week 4 almost completely abrogated MRGPRX2 expression which was also reflected on a functional level by reduced or absent SP and C48/80-mediated MC activation. Use of FBS should thus be taken into account in co-culture models involving MC. IL-6 is reported to improve MC yield and maturation and is thus widely used in MC culture protocols (48, 49). IL-6 had no noticeable impact on MRGPRX2 expression but did increase MC purity after 4 weeks, in line with results reported by Elst et al. (34).

Taken together, these findings highlight the potential pitfalls of MRGPRX2 assays using cultured human MC. Since even short-term variations in culture conditions can negatively influence functional MRGPRX2 expression. While the mechanism by which this suppression occurs remains unknown, FBS and/or IL-9 supplementation could also be leveraged as an alternative MRGPRX2 suppression/inhibition strategy to RNA silencing or pharmacological inhibitors (50).

Inter- and intra-donor variability is also an important factor since we and others observed that MC purity, phenotype and responsiveness can differ by several orders of magnitude between cultures (34–36, 39, 40, 51). Apart from potential technical variations inherent to the sensitive and long-term (up to several months) culture systems used for MC generation, intra-experimental variation between cultures derived from different donors and variation between cultures from the same donor performed at different timepoints suggest that donor-specific (epi)genetic factors as well as inter- and intra-donor variability in circulating MCP counts might play a role (4, 52). Donor age might be a determining factor since it negatively influences circulating CD34^+^ cell counts, which is reported to be a crucial factor in determining MC yield (37, 53). Another factor might be atopic status, since seasonal intra-donor variability in circulating MCP levels has been demonstrated (54). Other studies found no significant differences in MC cultured from atopic vs. non-atopic subjects (55–57). While we observed some inter-donor variability in MCP counts, our study was not designed to assess the impact of atopy or donor age on culture outcomes.

The speed of MC maturation also appears to differ between donors. We and others observed significant inter-donor variability in the percentage of mature CD117^+^_CD203c^+^ MC after 3-4 weeks of culture (34, 40, 58). Interestingly, intracellular MC protease expression at this stage, is already measurable in most cells, indicating that the CD117^-^ subpopulation likely consists of developing MC that have not yet reached maturity (34, 40). This is supported by studies with longer culture durations demonstrating > 90% MC purity at the end of the protocol (37, 38, 58). Lengthening culture duration could therefore reduce variability, albeit at the cost of increased material requirements, hands-on time and contamination risk.

Similar to other studies, we demonstrated functional IgE- and MRGPRX2-dependent activation pathways in MC cultured for 4 weeks (34, 40, 57). This enables use of these cells in the study of (non)-IgE-dependent hypersensitivity mechanisms (i.e. MAT). We opted for flow cytometry since it enables specific analysis of culture subpopulations (e.g. CD117^+^, MRGPRX2^+^) compared to supernatant/lysate-based assays (e.g. beta-hexosaminidase), an advantage in shorter culture protocols where some cells have yet to reach phenotypical maturity (25). Some authors propagate MAT as a viable alternative to the classic basophil activation test (BAT) due to the latter’s drawbacks (i.e. reliance on fresh samples, non-responders, lack of baseline MRGPRX2 expression) (25, 27, 51, 59). In our hands and compared to our BAT experience, maximum IgE-dependent CD63 expression was relatively low, in line with findings from other groups (28, 34, 40). Further optimization of our sensitization/stimulation steps using known MC priming factors such as SCF, IL-6 and/or the alarmin IL-33 might enhance IgE-dependent responses (41, 57).

We also delivered additional proof-of-concept for screening pharmacological compounds for their inhibitory potential on (non)-IgE-dependent MC activation. Interestingly, the MC stabilizing H1 antihistamine ketotifen was shown to directly inhibit both IgE- and MRGPRX2-dependent activation, expanding upon previous research and supporting its use in both IgE-dependent allergy as well as non-IgE-dependent disorders such as clonal MC disorders (60, 61). Nevertheless, our work does not reveal the mechanism by which this inhibition occurs and further research focusing on the impact of ketotifen on intracellular signaling downstream from stimulatory MC receptors is required. Contrasting with previous studies, we could not observe an impact of CS on MC activation (61). Notably, the mechanism of action of CS remains largely unknown and might rely on inhibition of non-MC cell types (62).

Regardless of the stimulation protocol used, MC donor-specific variability in receptor expression and agonist responses remain a hurdle in the clinical application of MAT. In our hands, MRGPRX2 expression on PB-derived MC varied between 0% and 70%. Similarly, human skin-derived MC also demonstrate significant inter-donor variability in MRGPRX2 expression, ranging from 0% to almost 100% (22, 33). Immortalized MC lines that express human FcϵRI and/or MRGPRX2 offer an alternative platform to primary cells. LAD-2 cells ubiquitously and stably express MRGPRX2 albeit at lower densities compared to PB-derived MC cultured for 12 weeks (28, 63). While LAD-2 cells express both FcϵRI and MRGPRX2, they exhibit slow growth, variable and/or weak IgE-responses and different MRGPRX2 kinetics from primary MC (25, 28, 51, 64). Hermans et al. demonstrated Latrunculin-B inducible functional MRGPRX2 expression on the fast-growing but dedifferentiated HMC-1 cell line (28). More recently, so-called Hoxb8 mast cells were described to proliferate rapidly and stably express high levels of functional FcϵRI, while lacking human MRGPRX2 expression (65). Of note, a clinical MAT-based test for MRGPRX2-mediated hypersensitivity reactions ideally uses a model with the same genetic background as the patient, since polymorphisms might determine individual susceptibility to MRGPRX2 agonists (27, 33–36).

Some limitations of our study include a lack of paired experiments in some conditions; lack of direct comparison between low- and high-density seeded BC-derived cultures which precludes us from definitively concluding an impact of sample type on culture yield; use of a short semi-solid medium-based culture model, limiting translatability to cultures with longer duration and/or liquid media; lack of mechanistic explanation regarding the observed impact of microenvironmental conditions and pharmacological inhibitors on MRGPRX2 expression and activation; exclusive reliance of CD63 as single marker of MC activation and autofluorescence preventing comparison of FcϵRI expression between various conditions.

In summary, our work demonstrates for the first time that functional MRGPRX2 expression on primary human mast cells cultured for 4 weeks is negatively influenced by specific culture conditions, including seeding densities above 1x10^5^ cells/mL as well as (short-term) exposure to IL-9 and FBS. Whenever primary human MC are generated for the MRGPRX2-dependent assays, culture conditions should be carefully optimized and validated.

## Data Availability

The datasets presented in this article are not readily available because of privacy restrictions. Data can be made available upon specific and reasonable request. Requests to access the datasets should be directed to toon.ieven@kuleuven.be.
