# Supplementary material for: Functional MRGPRX2 expression on peripheral blood-derived human mast cells increases at low seeding density and is suppressed by interleukin-9 and fetal bovine serum
Source: Front Immunol. 2024 Dec 13;15:1506034. doi: 10.3389/fimmu.2024.1506034 (PMC11683848; doi:10.3389/fimmu.2024.1506034)
Supplement: Supplementary file 1 [file DataSheet1.docx]

*Supplemental online material belonging to*

**Functional MRGPRX2 expression on peripheral blood-derived primary human mast cells is suppressed by high seeding densities and exposure to interleukin-9 or fetal bovine serum**

**Contents**

- Supplementary methods
  1. Cell counting (page 2)
  2. Progenitor isolation (page 2)
  3. Mast cell cultures (page 2)
  4. Stimulation and inhibition experiments (page 3)
  5. Immunophenotyping (page 3)
- Supplementary tables
  1. Table S1 (page 5)
  2. Table S2 (page 6)
- Supplementary figure captions (page 7)
- References (page 10)

**Supplementary methods**

1. **Cell counting**

Cell counts were obtained after manual counting under a light microscope using a Burker haemocytometer. Viability was assessed through trypan blue exclusion dye staining. All counts indicate viable/live cells only.

1. **Progenitor isolation (figure 1A)**

First, PB mononuclear cells (PBMC) were isolated from PB and BC using density gradient centrifugation with Lymphoprep^TM^ density medium (1.077 g/L; Stemcell Technologies). PBMC were assessed for viability (≥ 90%), counted and suspended at 1x10^8^ cells/mL in PBS - 2% fetal bovine serum (FBS) - 1mM EDTA and transferred to sterile 5 mL polystyrene tubes. Next, CD34^+^ progenitors were enriched through positive immunomagnetic selection using the EasySep^TM^ Human CD34 Positive Selection Kit II (Stemcell Technologies, Vancouver, Canada) according to the manufacturer’s instructions. A total of 5 selection cycles were performed for each sample, followed by suspension and counting of the positively selected PBMC fraction in 1 mL Iscove’s modified Dulbecco’s medium (IMDM; Gibco ThermoFisher, Waltham, Mass, U.S.).

1. **Mast cell cultures**

The culture protocol consisted of 3 phases, including a starting phase, nourishing phase and resting phase and was adapted from previously published reports with modifications (1-4). See **Figure S1** for culture protocol overview. All media were preheated to 37°C prior to contact with cells. Centrifugation of cultured MC was done at 300g for 5’ at room temperature (RT) unless otherwise stated (5).

- 1. Starting phase

Starting medium (SM) consisted of semi-solid methylcellulose-based serum-free medium (MethoCult^TM^, Stemcell Technologies), thawed slowly overnight and shaken vigorously before use, combined 4:1 with IMDM with supplements including penicillin-streptomycin (P/S; 100 U/mL-100 µg/mL), β-mercaptoethanol (55 µM), human low-density lipoprotein (LDL; 10 µg/mL, Stemcell Technologies), recombinant human SCF (100 ng/mL; Miltenyi Biotec, Bergisch Gladbach, Germany) and recombinant human interleukin (IL)-3 (50 ng/mL; PeproTech, Cranbury, NJ, U.S.). SM was mixed with the positively selected PBMC fraction at varying densities and vortexed for 5 seconds to distribute cells evenly. Medium was distributed in 6- or 24-well plates (1.1 mL/well) using a 5 mL syringe with a 16G blunt-end needle. Outside wells were filled with sterile water to maintain humidity. Culture plates were placed in a sterile incubator at 37°C and 5% CO_2_. Variations in starting conditions included low (1-4x10^4^/mL) or high (1-2x10^5^/mL) seeding densities and adding/withholding recombinant human IL-6 (50 ng/mL; PeproTech). Preliminary experiments revealed no benefit of higher cytokine concentrations (100 ng/mL; data not shown).

- 1. Nourishing phase

Cultures were nourished twice weekly by gently layering 0.3 mL nourishing medium (NM) on top of each well. NM consisted of IMDM supplemented with bovine serum albumin (BSA; 0.1%), P/S (100 U/mL-100 µg/mL), SCF (20 ng/mL), insulin-transferrin-selenium (ITS-G; 1x; Gibco) and various cytokines concentrated at 20 ng/mL. Variations in the nourishing phase included duration of IL-3 supplementation (week 1 vs. continuous), IL-6 supplementation (none vs. continuous) and addition of IL-9 (week 2-3).

- 1. Resting phase

After 3 weeks in MethoCult, cells were harvested after gently removing adherent cells with a cell scraper and thoroughly rinsing wells with preheated sterile PBS-BSA 0.5%. The remaining MethoCult was further diluted in PBS-BSA in a 1:4 ratio and centrifuged for 10’ at 300g followed by carefully removing the supernatant by aspiration without disturbing the pellet. Cells were counted and resuspended at a concentration of 0.5x10^6^/mL in resting medium (RM) consisting of IMDM with SCF (20 ng/mL) and P/S (100 U/mL-100 µg/mL), modified by adding/withholding FBS 5%. Cells were divided across 16-well plates (1.1 mL/well) and placed in a sterile incubator at 37°C and 5% CO_2_ until harvesting 3-7 days later and resuspension in appropriate assay buffers at 0.25-0.5x10^6^/mL for final analysis.

1. **Stimulation and inhibition experiments**

For each condition, 25.000-50.000 cells were suspended in 90 µL HEPES buffer (6). Stimulants and inhibitors were acquired from Sigma-Aldrich (St. Louis, MO, U.S.), stored at -20°C and diluted in buffer to 10X and 100X working solutions, respectively. HEPES buffer was used as negative control. Indicated concentrations are final concentrations in the total reaction volume.

For IgE-mediated stimulation, cells were passively sensitized overnight at 37°C and 5% CO_2_ with serum of a single donor added to RM at a 1:10-1:2 ratio. The following day, cells were washed twice in HEPES to remove serum, followed by stimulation with 10 µL polyclonal goat anti(a)-IgE (1 µg/mL) for 60’ at 37°C and resting on ice for 5’.

For MRGPRX2-mediated stimulation, unsensitized cells were incubated with either 10 µL substance P (SP; 0.1 mg/mL or 74.2 µM) or compound 48/80 (C48/80; 3.12 µg/mL or 0.62 µM) for 20’ at 37°C followed by resting on ice for 5’.

For inhibition experiments, cells were incubated prior to stimulation with 1 µL of buffer or various inhibitors, including dasatinib (das; 1 µM), ketotifen (ket; 100 µM) and cromolyn sodium (CS; 1 mM) for 15’ at 37°C in a water bath followed immediately by stimulation.

After stimulation, cells were washed, and CD63 expression was assessed via flow cytometry to measure activation (7).

1. **Immunophenotyping**

Dead cells were first stained using Fixable Viability Dye eFluor^TM^ 780 according to the manufacturer’s instructions (FVD780; eBioscience, San Diego, Calif., U.S.) followed by washing, staining in the dark for 25’ at 4°C with appropriate fluorochrome-conjugated antibody panels (see **Table S1**), washing and fixation in paraformaldehyde (PFA) 1% and storage at 4°C prior to acquisition on an LSR Fortessa flow cytometer equipped with FACSDiva software (BD, Franklin Lakes, NJ, U.S.). Fluorochrome-conjugated antibodies were acquired from BioLegend (San Diego, Calif., U.S.). Final analysis was performed using FlowJo v10.8.1 for Windows (BD, San Jose, Calif., U.S.). Full sets of compensation controls (UltraComp eBeads^TM^ Plus; Invitrogen; Waltham, Mass., U.S.) were included for each experiment and fluorescence-minus-one (FMO) controls for initial experiments. Inactivated human plasma and Brilliant stain buffer (eBioscience) were used to block non-specific binding and allow for combination of brilliant violet stains, respectively.

For progenitor characterization, positively and negatively selected PBMC fractions (100.000-200.000 cells) were washed and stained. Progenitors were identified as CD34^+^_CD45^-^ and MC-committed progenitors (MCP) were identified as CD34^+^_CD117^+^_FcεRIα^+^ as previously reported (see **Figure S2** for gating strategy) (8).

To assess differentiation and activation, cultured cells were harvested and stained (25.000-50.000 cells per condition). Mature MC were identified as CD117^+^_CD203c^+^ and the %MRGPRX2^+^ MC was assessed within this population (see **Figure S3** for gating strategy). MC activation was evaluated by measuring %CD63^+^ cells within the total MC and MRGPRX2^+^ MC populations (see **Figure S3** for gating strategy). The final %CD63^+^ MC was calculated by subtracting percentages in the unstimulated (buffer) from the stimulated conditions. Stimulation indices (SI) were calculated as the ratio of %CD63^+^ cells in the stimulated over unstimulated conditions.

Tryptase and chymase expression were assessed through intracellular (IC) staining. In brief, after viability staining, 5-10x10^5^ cells were fixated and permeabilized with appropriate buffers (eBioscience) according to the manufacturer’s instructions. Next, primary mouse aTryptase and rabbit aChymase (Abcam, Cambridge, U.K.) were added, followed by washing and addition of secondary donkey aMouse (AF488) and donkey aRabbit (AF594), incubation for 30’ at 4°C followed by washing and fixation in PFA 1% (see **Figure S4** for gating strategy).

**Supplementary tables**

| **panel** | **# cells/sample** | **markers** | **fluorochrome** | **volume/sample** |
| --- | --- | --- | --- | --- |
| Progenitor | 1-2 x 10^5^ | FVD (viability) | eFluor780 | 1 mL (1:10.000 dilution) |
|  |  | CD34 | FITC | 1 µL |
|  |  | CD45 | V500 | 1 µL |
|  |  | CD117 | BV711 | 1 µL |
|  |  | FcεRIα | PE-Dazzle 594 | 0.5 µL |
| Differentiation / Activation | 2.5-5 x 10^4^ | FVD (viability) | eFluor780 | 1 mL (1:10.000 dilution) |
|  |  | CD117 | BV711 | 1 µL |
|  |  | CD203c | BV421 | 2 µL |
|  |  | MRGPRX2 | APC | 1 µL |
|  |  | FcεRIα | PerCP-Cy5.5 | 1 µL |
|  |  | CD63 | PE | 0.1 µL |
| Protease expression | 5-10 x 10^5^ | FVD (viability) | eFluor780 | 1 mL (1:10.000 dilution) |
|  |  | mouse anti-Tryptase (primary Ab) | anti-mouse AF488 (secondary Ab) | 1:100 in perm. buffer (for both) |
|  |  | rabbit anti-chymase (primary Ab) | anti-rabbit AF594 (secondary Ab) | 1:100 in perm. buffer (for both) |

**Supplementary table 1.** Fluorochrome-conjugated antibody panels used for immunophenotyping experiments.

| **Buffy coat concentrate (BC) donors** | | | | |
| --- | --- | --- | --- | --- |
| **ID** | **Age (y)** | **Sex** | **N cultures** | **Remarks** |
| ***All*** | *46.7* | *62.5% F* | *14** | *4 cultures failed* |
| **RK1** | n.a. | F | 1 | - |
| **RK2** | 29 | M | 1* | culture failed due to contamination |
| **RK3** | 49 | M | 1 | - |
| **RK4** | 61 | F | 1 | - |
| **RK5** | 57 | F | 1 | - |
| **RK6** | 66 | F | 3* | 3 cultures: 2 from stored PBMC, 1/2 failed |
| **RK7** | 26 | M | 1* | culture failed, PBMC used > 24h after donation |
| **RK8** | 30 | F | 1* | culture failed; progenitor characterisation |
| **RK9** | 66 | n.a. | 1 | progenitor characterisation |
| **RK10** | 36 | n.a. | 1 | progenitor characterisation |
| **Peripheral whole blood (PB) donors** | | | | |
| **ID** | **Age (y)** | **Sex** | **N cultures** | **Remarks** |
| ***All*** | *31.8* | *46.2% F* | *19* | *-* |
| **CO1** | 30 | M | 4 | progenitor characterisation, IC staining |
| **CO2** | 27 | F | 5 | IC staining |
| **CO3** | 28 | M | 1 | - |
| **CO4** | 29 | F | 1 | - |
| **CO5** | 27 | F | 1 | - |
| **CO6** | 29 | M | 1 | IC staining |
| **CO7** | 26 | M | 2 | progenitor characterisation |
| **CO8** | 25 | F | 1 | - |
| **CO9** | 30 | F | 1 | - |
| **CO10** | 32 | M | 1 | - |
| **CO11** | 54 | F | 0 | progenitor characterisation |
| **CO12** | 39 | M | 0 | progenitor characterisation |
| **CO13** | 38 | M | 1 | - |

**Supplementary table 2.** Overview of donors and experiments. * indicates presence of failed culture runs; F, female; M, male; IC, intracellular.

**Supplementary figure captions**

**Figure S1**. Mast cell purity (% CD117^hi^_CD203c^hi^) in peripheral blood-derived mast cells (MC) cultured for 4 weeks in MethoCult under varying culture conditions. **(A)** Overview of experimental culture conditions. **(B)** Unpaired comparison between buffy coat concentrate (BC)-derived and peripheral whole blood (PB)-derived MC cultures. **(C)** Paired comparison between BC-derived MC cultures seeded at high density (1-2 x10^5^ cells/mL) or low density (1-4 x10^4^ cells/mL). **(D)** Unpaired comparison between MC cultured in continuous presence of interleukin (IL)-3 or IL-3 during week 1 and IL-6 continuously. **(E)** Paired comparison between MC cultured in IL-3 and IL-6 or IL-3 and IL-6 with addition of IL-9 during week 2. **(F)** Paired comparison between MC cultured with or without fetal bovine serum (FBS) during week 4. Red dots correspond to exemplary flow cytometry plots shown. Error bars show median and interquartile ranges for unpaired data. For paired data, cultures obtained from the same donor during the same experiment are connected with lines. Horizontal lines indicate comparisons between groups using the Mann-Whitney U test for unpaired data and the Wilcoxon matched-pairs signed rank test for paired data. Pairwise comparisons were made between cultures derived from the same donor during the same culture run. ns = not significant (p > 0.05), * = p < 0.05.

**Figure S2.** Representative gating strategy for characterisation of starting samples after positive immunomagnetic CD34^+^ selection. **(A)** Gating on positively selected (CD34^+^ enriched) PBMC fraction. **(B)** Gating on negatively selected PBMC fraction (i.e. rest fraction discarded after successive positive selection steps). Gates for CD34, CD45, CD117 and FcεRIα were set based on fluorescence-minus-one (FMO) controls performed for each experiment. Data shown from a peripheral whole blood donor (CO1). Black arrows indicate directionality of flowcytometric gating. Progenitors, i.e. peripheral blood stem cells (PBSC) - were identified as CD34^+^_CD45^-^ and MC-committed progenitors (MCP) were identified within this population as CD117^+^_FcεRIα^+^ (top right quadrant in final panel)

**Figure S3.** Representative gating strategy for characterisation of cultured cells after 4 weeks. **(A)** Gating of mast cells (MC; CD117^+^_CD203c^hi^) within live single cells. Mast cells were assessed separately for MRGPRX2 and FcεRIα expression. Due to autofluorescence, no clear positive/negative FcεRIα gate could be set. CD117, CD203c and MRGPRX2 gates were set based on fluorescence-minus-one controls (FMO) performed in initial selected experiments. **(B)** Gating of activated (CD63^+^) mast cells within total MC (left panels) and within MRGPRX2^+^ MC (right panels). CD63^+^ gate was set to exclude the largest population in each experiment’s corresponding buffer (negative) control condition. Data shown from a representative culture derived from a peripheral whole blood donor (CO2, experiment MC10). Black arrows indicate directionality of flowcytometric gating.

**Figure S4.** Representative gating strategy for flow cytometric assessment of intracellular mast cell protease (tryptase and chymase) expression. Tryptase and chymase gates were set based on fluorescence-minus-one controls (FMO) performed for each experiment. Black arrows indicate directionality of flowcytometric gating.

**Figure S5.** Peripheral blood mononuclear cell (PBMC) and positive selection yields normalized per **(A)** mL starting sample (i.e. 40 mL buffy coat concentrate (BC) or 80 mL peripheral whole blood (PB)) or per **(B)** mL initial donor blood volume (i.e. 500 mL for BC and 80 mL for PB). Horizontal bars indicate results of Mann-Whitney U unpaired comparisons between groups. ns = not significant (p > 0.05), **** = p < 0.001.

**Figure S6.** Peripheral blood mononuclear cell (PBMC) yields after density gradient centrifugation normalized per mL initial donor blood volume according to sample type (buffy coat concentrate (BC) vs. peripheral whole blood (PB)), donor sex (male (M), female (F)) and donor age (with linear regression line). Horizontal error bars indicate results of Mann-Whitney U unpaired comparisons between groups. ns = not significant (p > 0.05).

**Figure S7**. **(A)** Relationship between total amount of seeded cells and final culture yield (i.e. total live cells harvested from cultures after 4 weeks) stratified according to seeding density. Linear regression lines with slope and 95% confidence intervals are shown for low-density seeded culture yields (dotted line, open circles) and high-density seeded culture yields (full line, closed circles). **(B)** Culture yield expressed as ratio of total harvested cells at 4 weeks to total seeded cells at day 0 for high-density (Hi D, closed circles) and low-density seeded cultures (Lo D, open circles). Horizontal error bar indicates results of Mann-Whitney U comparison between groups. ** = p < 0.01.

**Figure S8.** Mast cell (MC) activation test with yellow jacket venom (YJV). CD63 dose-responses curve after overnight sensitization with human serum (1:10 – 1:2) and stimulation with yellow jacket venom (YJV; 0,01 µg/mL – 10 µg/mL). MC activation is reported as % CD63+ live MC corrected for spontaneous activation (left y-axes, continuous lines) or as CD63 stimulation indices (SI; right y-axes, dotted lines). Experiments were performed on mast cells obtained from a single donor (CO1, MC14) after 4 weeks of culture in MethoCult with IL-3 (week 1) and IL-6 (continuously). Serum was obtained from a single yellow jacket venom allergic donor, different from the MC donor.

**Figure S9.** Phenotype and stimulus responses of peripheral blood-derived mast cells (MC) cultured with or without fetal bovine serum (FBS) during the final week. All MC were obtained after 4 weeks of culture in MethoCult with IL-3 (week 1) and IL-6 (continuous) without (- FBS) or with (+ FBS) supplementation of medium with FBS 5% during the final week. Y-axis shows % live MC (green diamonds) and % MRGPRX2^+^ MC (blue triangles) obtained at the end of the culture and % CD63^+^ MC corrected for spontaneous activation (red circles) after stimulation with substance P 0,1 mg/mL, compound 48/80 3,12 µg/mL or polyclonal goat anti-human IgE 10 µg/mL (after overnight passive sensitization with human serum 1:5).

**Figure S10.** Paired responses of peripheral blood-derived primary human mast cells (MC) to various stimuli and inhibitors. All MC were obtained after 4 weeks culture in MethoCult with IL-3 (week 1) and IL-6 (continuous). MC activation is reported as % CD63^+^ MC corrected for spontaneous activation (left y-axes, continuous lines). Pairwise comparisons were made between samples derived from the same donor during the same culture run. **(A)** Paired CD63 responses after preincubation with inhibitors and subsequent stimulation with substance P (SP) 0,1 mg/mL (n=8 donors). **(B)** Paired CD63 responses after preincubation with inhibitors and subsequent stimulation with compound (C) 48/80 3,12 µg/mL (n=5 donors), **(C)** Paired CD63 responses after overnight passive sensitization with human serum 1:5, preincubation with inhibitors and subsequent stimulation with anti-IgE 10 µg/mL. Horizontal bars indicate pairwise comparisons between groups using the Wilcoxon matched-pairs signed rank test. ns = not significant (p > 0.05).

**References**

1. Saito H, Kato A, Matsumoto K, Okayama Y. Culture of human mast cells from peripheral blood progenitors. Nat Protoc. 2006;1(4):2178-83. doi: 10.1038/nprot.2006.344.
2. Lappalainen J, Lindstedt KA, Kovanen PT. A protocol for generating high numbers of mature and functional human mast cells from peripheral blood. Clin Exp Allergy. 2007 Sep;37(9):1404-14. doi: 10.1111/j.1365-2222.2007.02778.x.
3. Schmetzer O, Valentin P, Smorodchenko A, Domenis R, Gri G, Siebenhaar F, et al. A novel method to generate and culture human mast cells: Peripheral CD34+ stem cell-derived mast cells (PSCMCs). J Immunol Methods. 2014 Nov;413:62-8. doi: 10.1016/j.jim.2014.07.003.
4. Cop N, Decuyper II, Faber MA, Sabato V, Bridts CH, Hagendorens MM, et al. Phenotypic and functional characterization of in vitro cultured human mast cells. Cytometry B Clin Cytom. 2017 Sep;92(5):348-354. doi: 10.1002/cyto.b.21399.
5. Hermans MAW, van Stigt AC, van de Meerendonk S, Schrijver B, van Daele PLA, van Hagen PM, van Splunter M, Dik WA. Human Mast Cell Line HMC1 Expresses Functional Mas-Related G-Protein Coupled Receptor 2. Front Immunol. 2021 Mar 15;12:625284. doi: 10.3389/fimmu.2021.625284.
6. Kuehn HS, Radinger M, Gilfillan AM. Measuring mast cell mediator release. Curr Protoc Immunol. 2010 Nov;Chapter 7:Unit7.38. doi: 10.1002/0471142735.im0738s91.
7. Knol EF, Mul FP, Jansen H, Calafat J, Roos D. Monitoring human basophil activation via CD63 monoclonal antibody 435. J Allergy Clin Immunol. 1991 Sep;88(3 Pt 1):328-38. doi: 10.1016/0091-6749(91)90094-5.
8. Dahlin JS, Malinovschi A, Öhrvik H, Sandelin M, Janson C, Alving K, et al. Lin- CD34hi CD117int/hi FcεRI+ cells in human blood constitute a rare population of mast cell progenitors. Blood. 2016 Jan 28;127(4):383-91. doi: 10.1182/blood-2015-06-650648.
