# Supplementary figures and images for: Functional MRGPRX2 expression on peripheral blood-derived human mast cells increases at low seeding density and is suppressed by interleukin-9 and fetal bovine serum
Source: Front Immunol. 2024 Dec 13;15:1506034. doi: 10.3389/fimmu.2024.1506034 (PMC11683848; doi:10.3389/fimmu.2024.1506034)

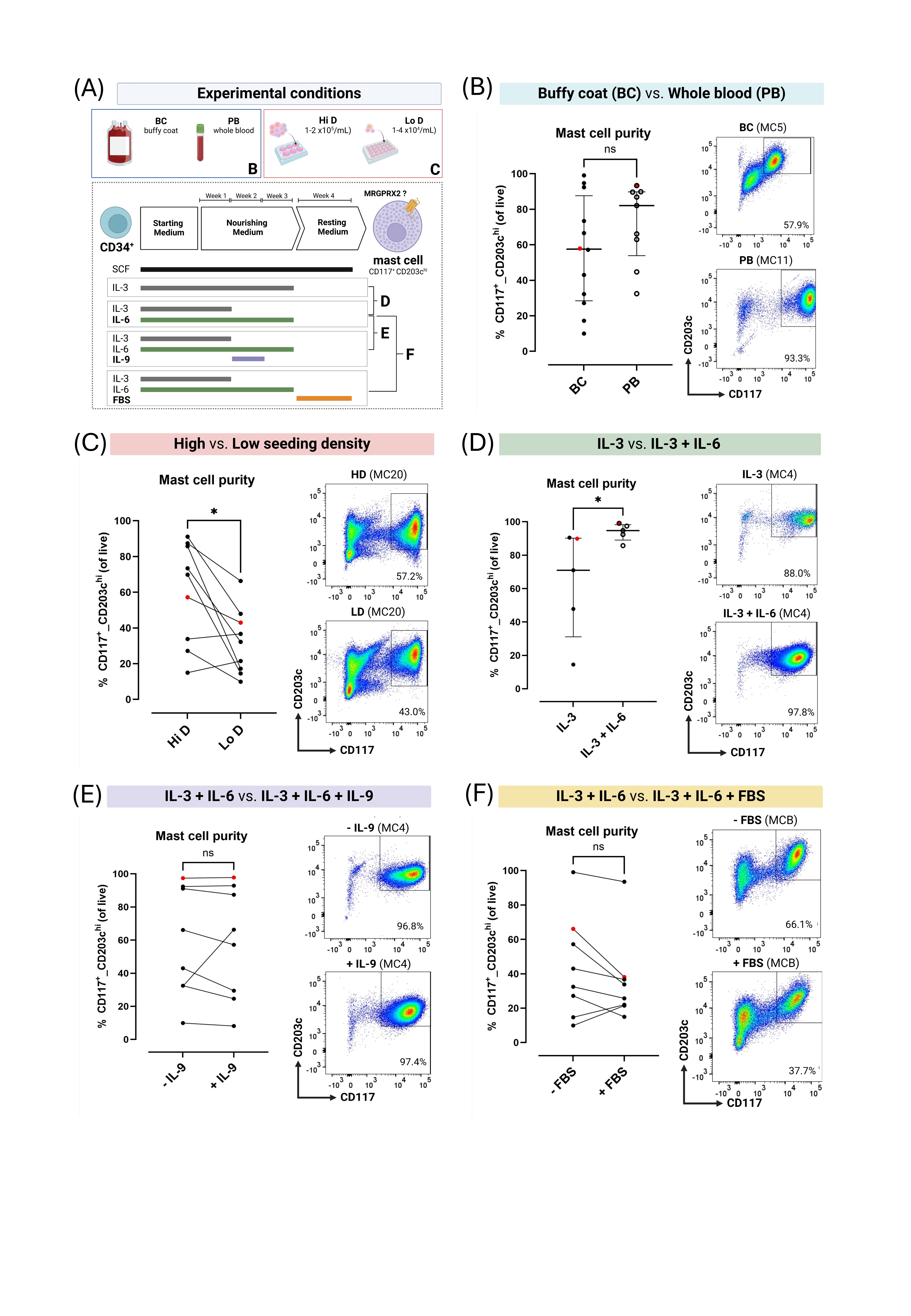

Supplement: Supplementary file 2 [file Image1.jpeg]

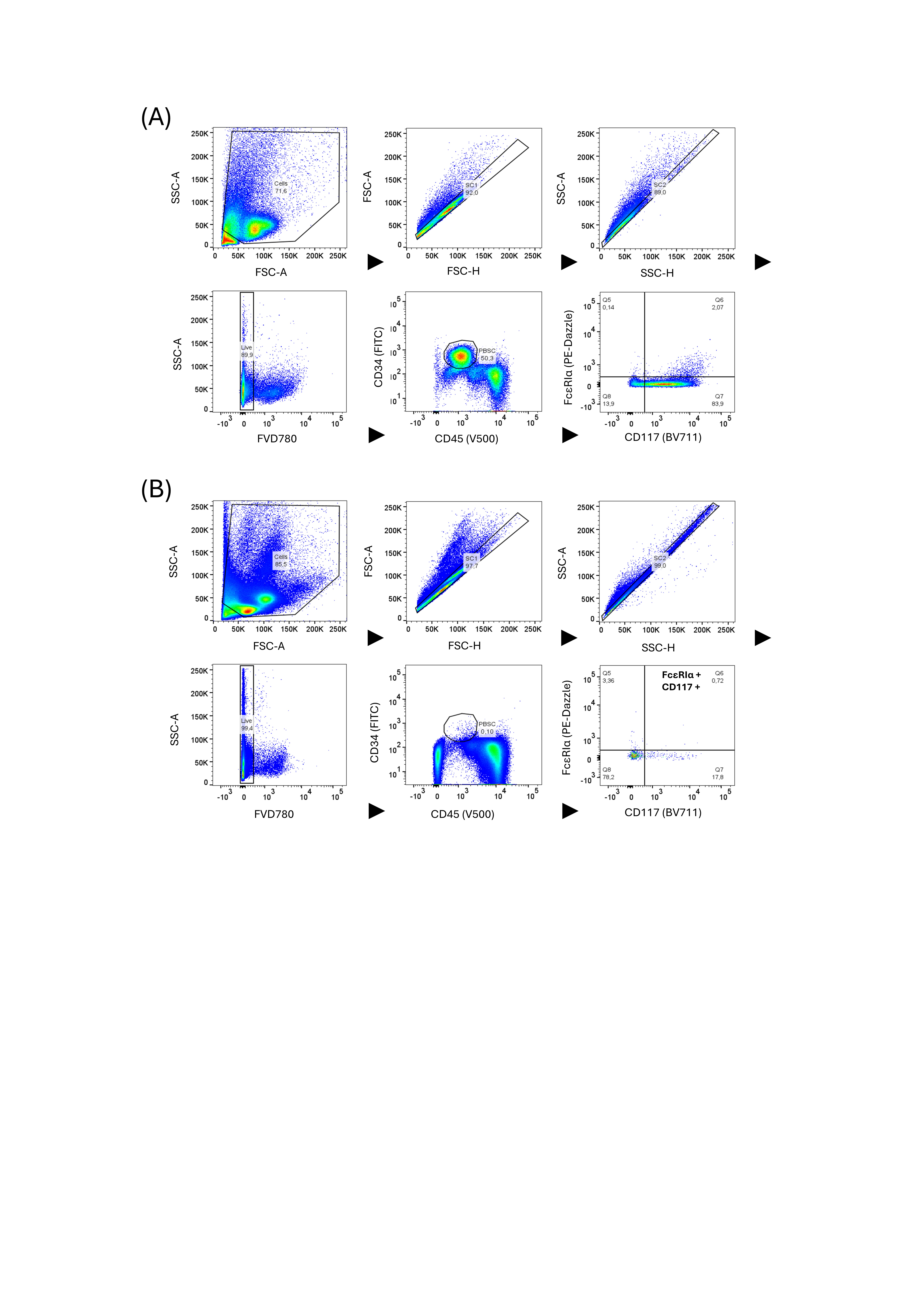

Supplement: Supplementary file 3 [file Image2.jpeg]

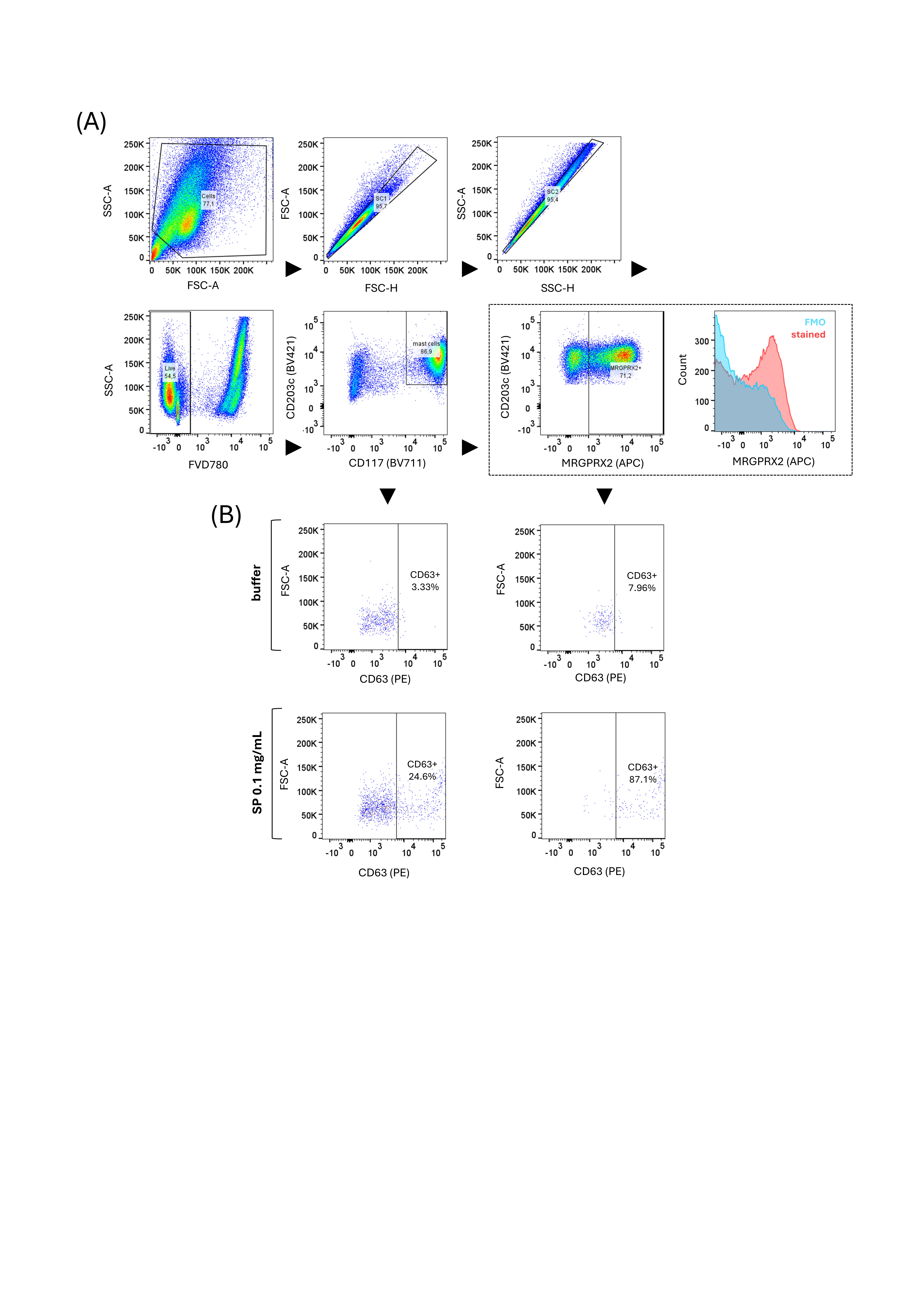

Supplement: Supplementary file 4 [file Image3.jpeg]

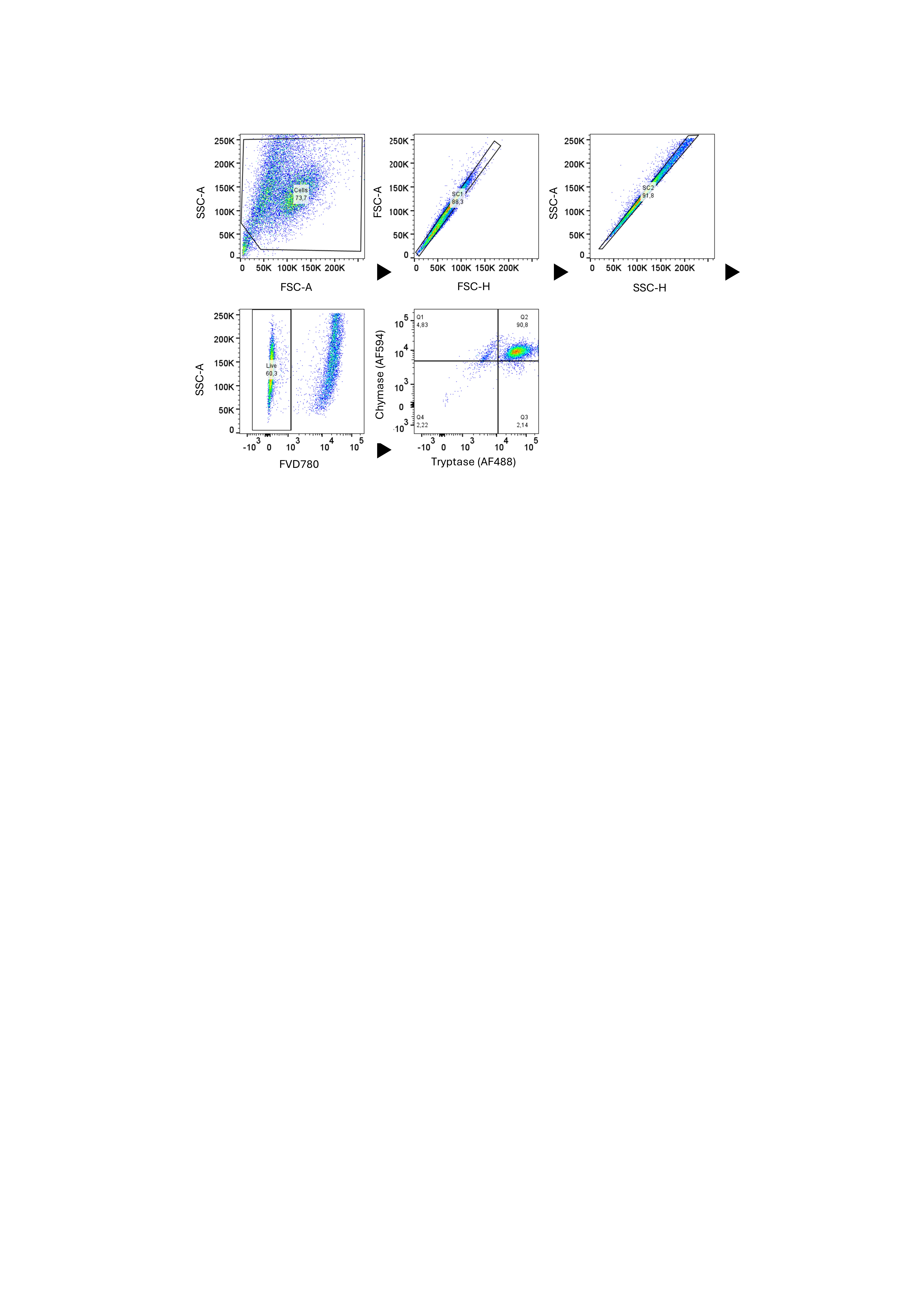

Supplement: Supplementary file 5 [file Image4.jpeg]

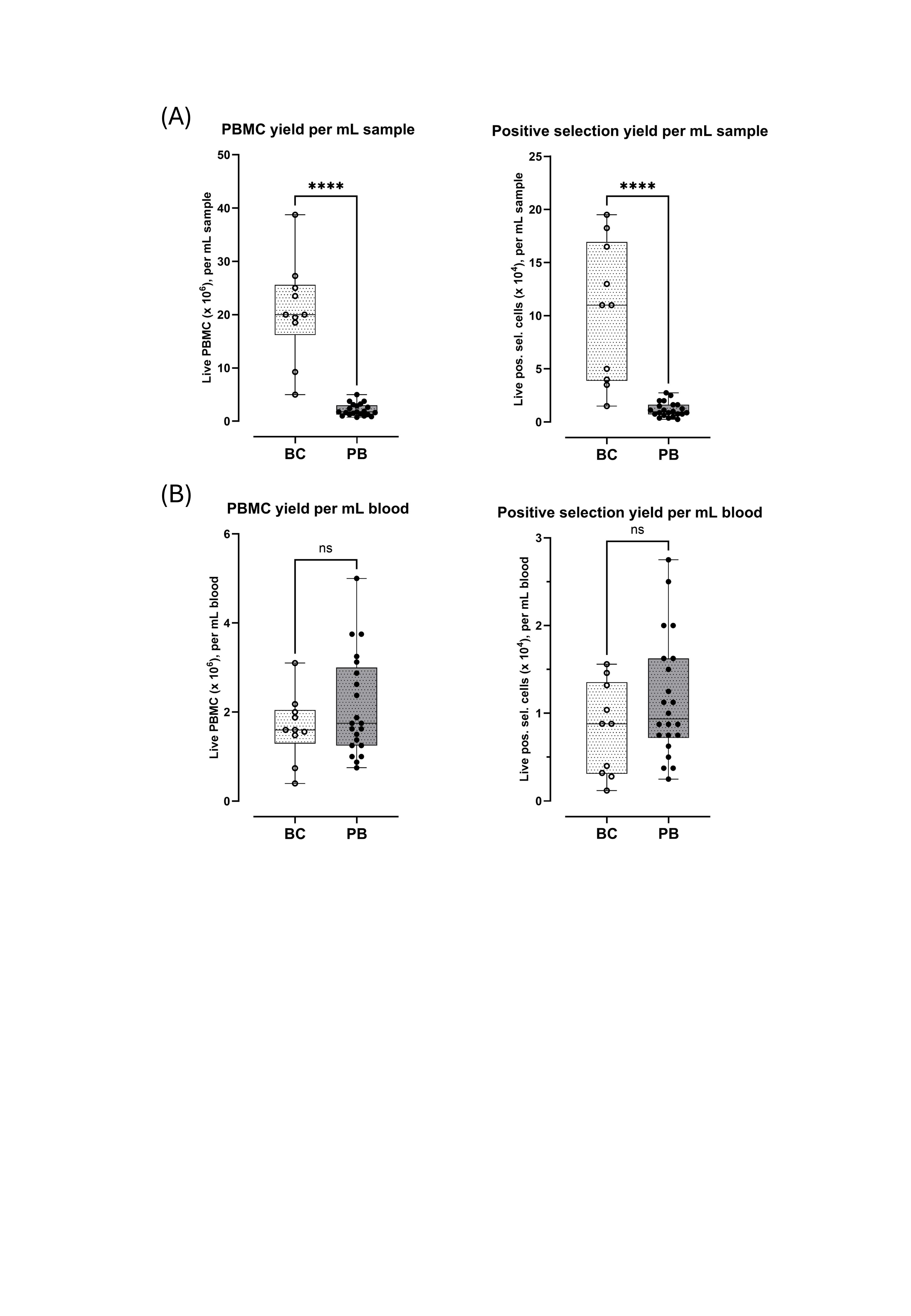

Supplement: Supplementary file 6 [file Image5.jpeg]

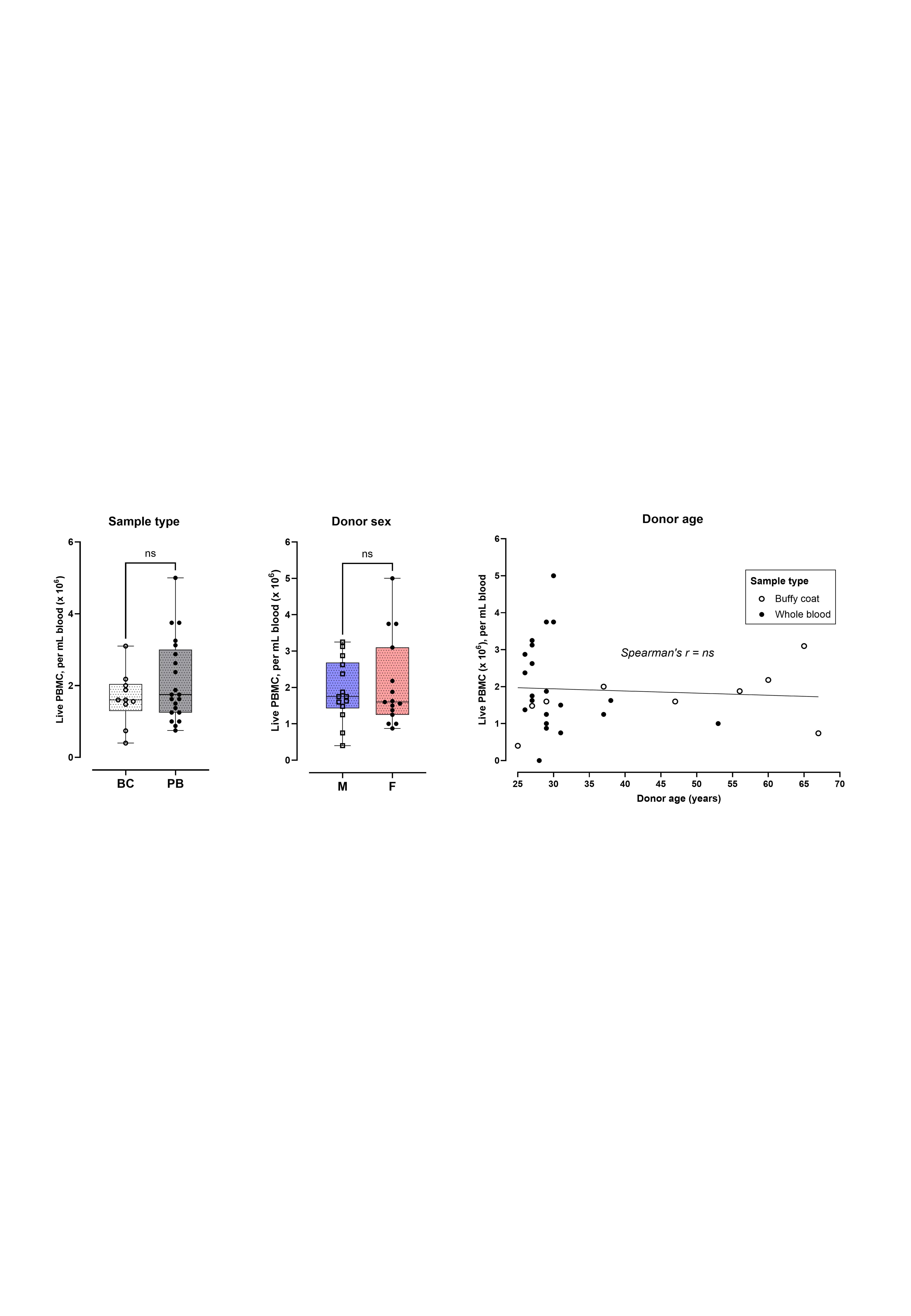

Supplement: Supplementary file 7 [file Image6.jpeg]

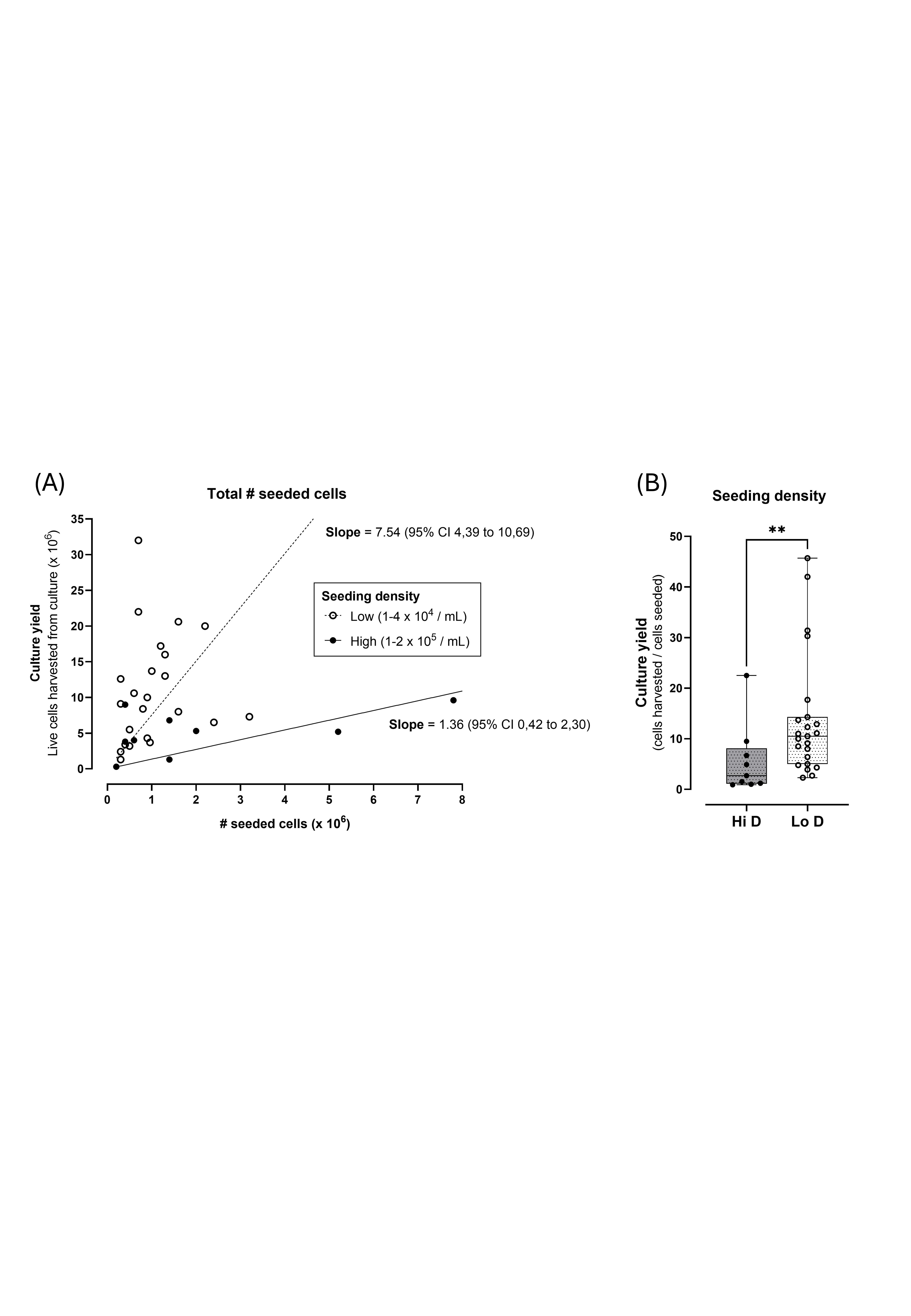

Supplement: Supplementary file 8 [file Image7.jpeg]

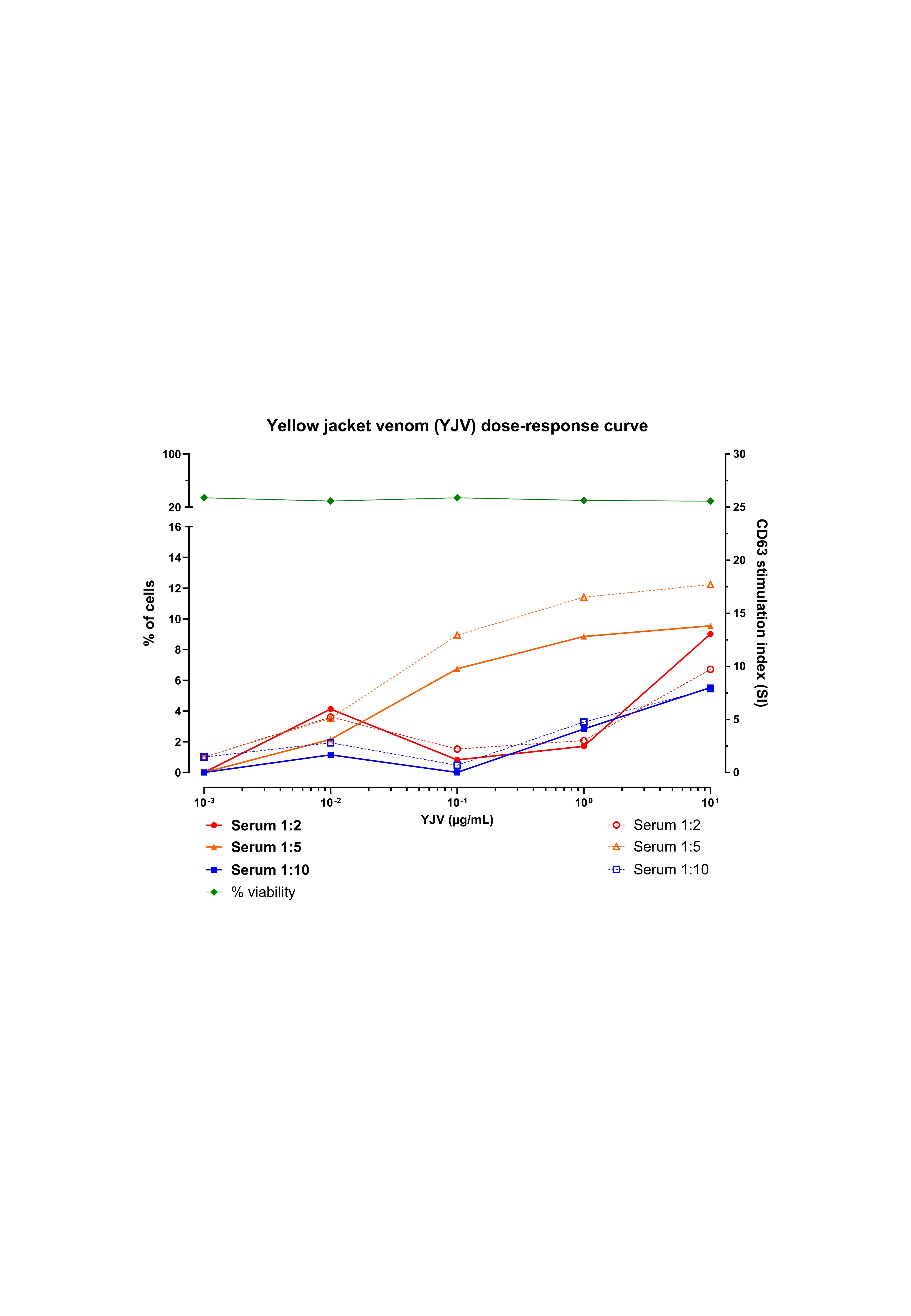

Supplement: Supplementary file 9 [file Image8.jpeg]

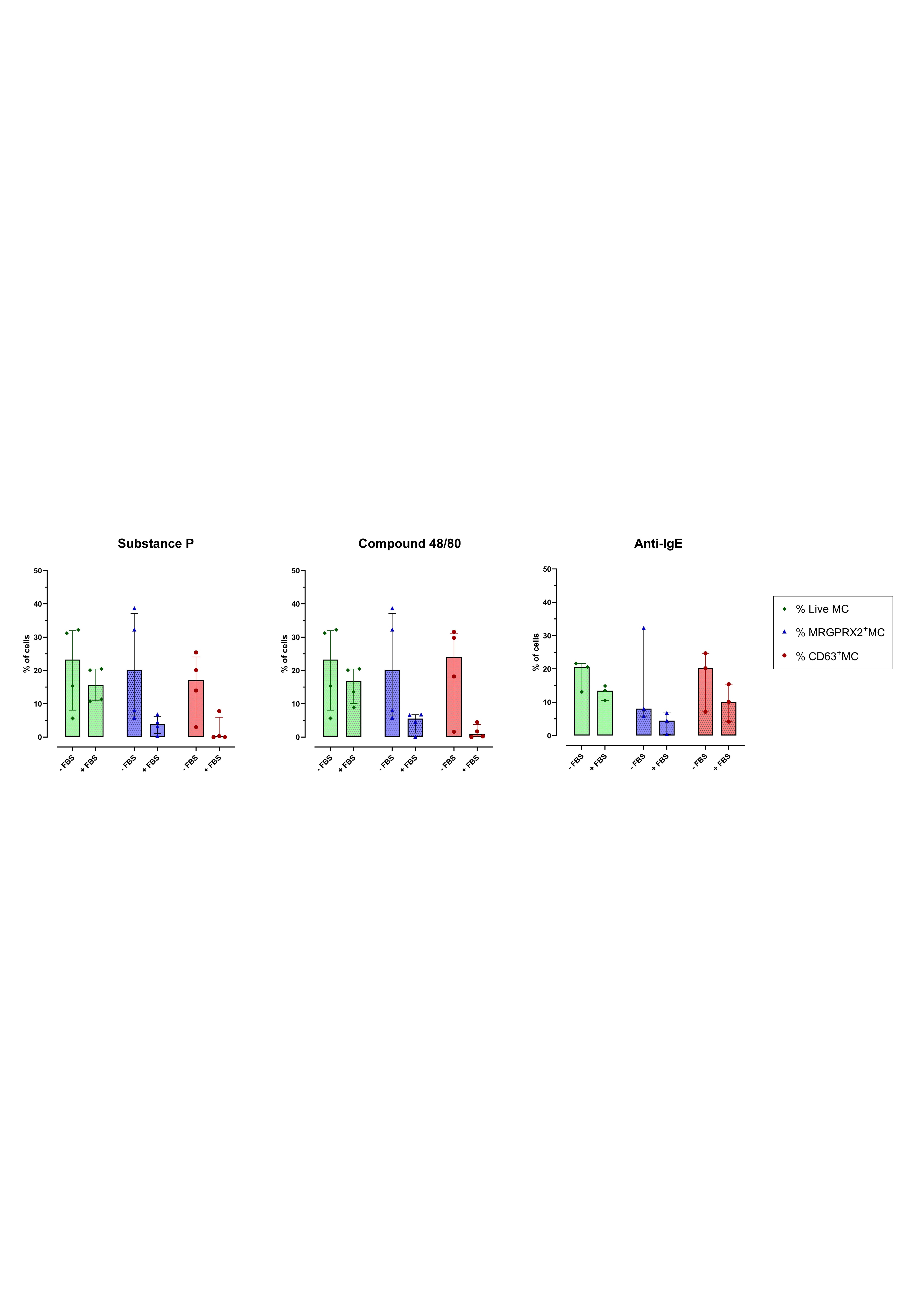

Supplement: Supplementary file 10 [file Image9.jpeg]

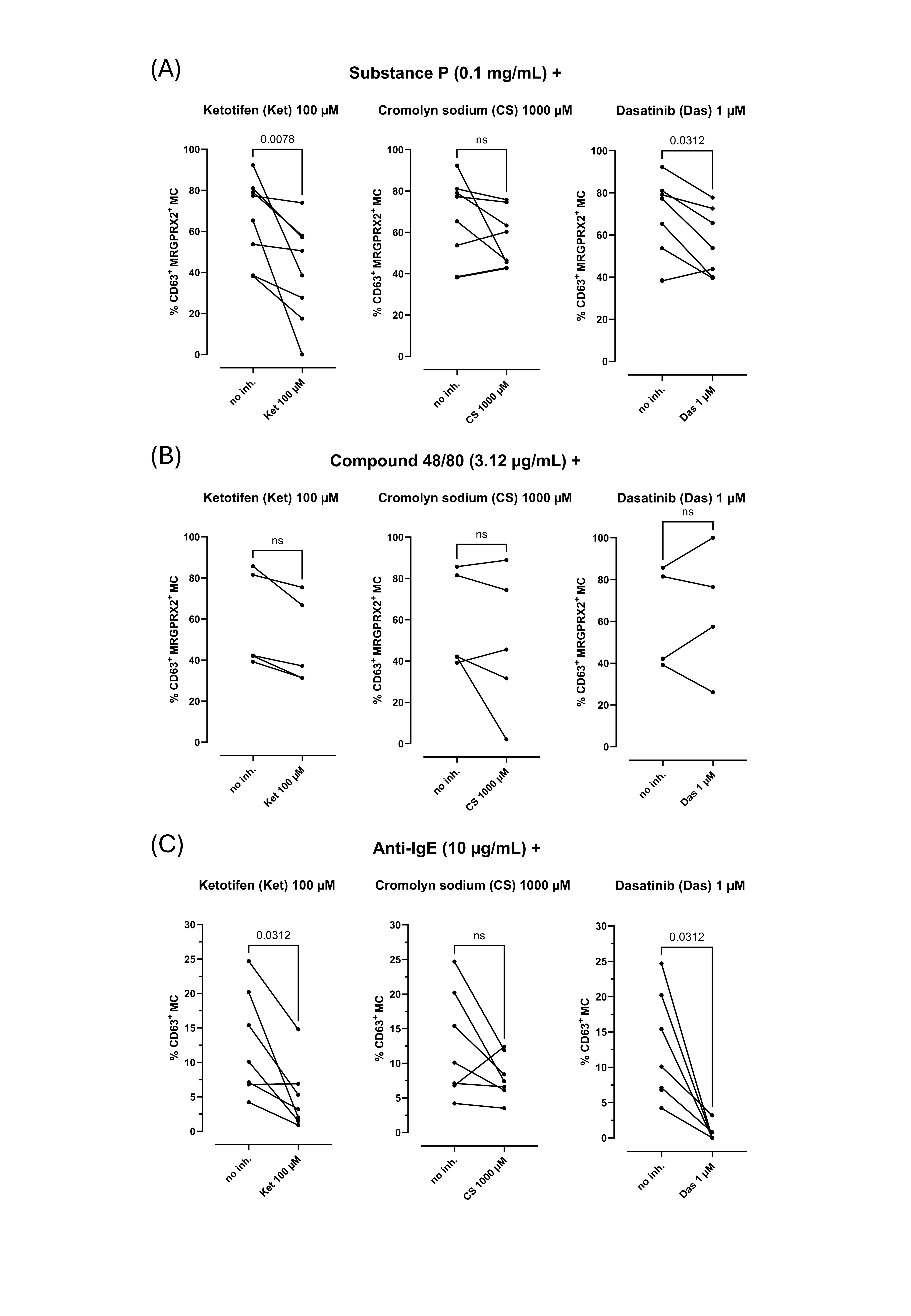

Supplement: Supplementary file 11 [file Image10.jpeg]
